# Suppression of eEF2 phosphorylation alleviates synaptic failure and cognitive deficits in mouse models of Down syndrome

**DOI:** 10.1002/alz.13916

**Published:** 2024-06-27

**Authors:** Xin Wang, Qian Yang, Xueyan Zhou, C. Dirk Keene, Alexey G. Ryazanov, Tao Ma

**Affiliations:** ^1^ Department of Internal Medicine Gerontology and Geriatric Medicine Wake Forest University School of Medicine Winston‐Salem North Carolina USA; ^2^ Department of Pathology University of Washington School of Medicine Seattle Washington USA; ^3^ Department of Pharmacology Rutgers Robert Wood Johnson Medical School Piscataway New Jersey USA; ^4^ Department of Translational Neuroscience Wake Forest University School of Medicine Winston‐Salem North Carolina USA

**Keywords:** cognition, Down syndrome, eukaryotic elongation factor 2 kinase, mouse model, protein synthesis, synaptic plasticity

## Abstract

**INTRODUCTION:**

Cognitive impairment is a core feature of Down syndrome (DS), and the underlying neurobiological mechanisms remain unclear. Translation dysregulation is linked to multiple neurological disorders characterized by cognitive impairments. Phosphorylation of the translational factor eukaryotic elongation factor 2 (eEF2) by its kinase eEF2K results in inhibition of general protein synthesis.

**METHODS:**

We used genetic and pharmacological methods to suppress eEF2K in two lines of DS mouse models. We further applied multiple approaches to evaluate the effects of eEF2K inhibition on DS pathophysiology.

**RESULTS:**

We found that eEF2K signaling was overactive in the brain of patients with DS and DS mouse models. Inhibition of eEF2 phosphorylation through suppression of eEF2K in DS model mice improved multiple aspects of DS‐associated pathophysiology including *de novo* protein synthesis deficiency, synaptic morphological defects, long‐term synaptic plasticity failure, and cognitive impairments.

**DISCUSSION:**

Our data suggested that eEF2K signaling dysregulation mediates DS‐associated synaptic and cognitive impairments.

**Highlights:**

- Phosphorylation of the translational factor eukaryotic elongation factor 2 (eEF2) is increased in the Down syndrome (DS) brain.
- Suppression of the eEF2 kinase (eEF2K) alleviates cognitive deficits in DS models.
- Suppression of eEF2K improves synaptic dysregulation in DS models.
- Cognitive and synaptic impairments in DS models are rescued by eEF2K inhibitors.

## 1 BACKGROUND

Down syndrome (DS), also known as trisomy 21 due to its association with the triplication of the chromosome 21 (HSA21), is the most common cause of intellectual disability.^1^, ^2^ Cognitive impairment including dementia is a core feature of DS and the leading cause of dependence in people with DS.^3^ Improvement of social and medical conditions over the past few decades has led to a significant increase in life expectancy for people with DS. Consequently, age‐related cognitive deficits rise markedly in DS. Interestingly, age‐related cognitive deficits in people with DS usually exhibit as dementia syndromes resembling Alzheimer's disease (AD), the most common form of dementia in the elderly and a devastating neurodegenerative disease.^4^, ^5^, ^6^, ^7^ Currently there is no effective treatment to improve cognitive defects in DS, and the neurobiological mechanisms underlying DS‐associated cognitive impairment remain unclear, hampering development of novel therapeutics.

Maintenance of long‐term synaptic plasticity and memory requires integral protein synthesis (mRNA translation) capacity.^8^, ^9^, ^10^ Recent studies indicate a role of translation dysregulation in multiple neurological disorders characterized by cognitive impairments including AD and DS.^11^, ^12^, ^13^, ^14^, ^15^ Overall protein synthesis includes three stages (initiation, elongation, and termination), with specific translational factors involved in each phase for accurate translational control to maintain cellular homeostasis under various physiological and pathological conditions.^10^ More than 95% of the energy and amino acids consumed in the mRNA translation are dedicated to the elongation phase.^16^, ^17^ Mounting evidence suggests that elongation regulation is particularly important during neuronal responses to deficiency of energy and nutrients.^8^, ^18^ Elongation is primarily regulated through phosphorylation of the eukaryotic elongation factor 2 (eEF2), which catalyzes movement of tRNA from the ribosomal A‐site to the P‐site via guanosine triphosphate hydrolysis.^19^ Phosphorylation of eEF2 on Thr^56^ by its (only known) kinase eEF2K results in disruption of peptide growth and thus inhibition of general protein synthesis.^20^, ^21^ Studies from both neuronal and non‐neuronal model systems identify multiple signaling cascades interacting with the eEF2K–eEF2 signaling including the mammalian (or mechanistic) target of rapamycin complex 1 (mTORC1) and the adenosine monophosphate–activated protein kinase (AMPK).^22^, ^23^, ^24^ Hyperphosphorylation of eEF2 has been demonstrated in AD brain samples, and we reported recently that suppression of eEF2K and eEF2 phosphorylation could alleviate synaptic failure and cognitive deficits in AD model mice without affecting the brain amyloid beta (Aβ) pathology.^25^, ^26^ How eEF2 phosphorylation and translation elongation control are involved in DS pathophysiology is unknown. The current study, mainly by using rodent models of DS, aims to test the central hypothesis that eEF2K–eEF2 signaling dysregulation plays an important role in DS‐associated cognitive deficits and synaptic failure with aging.

**RESEARCH IN CONTEXT**

1. **Systematic review**: Cognitive impairment including dementia is a core feature of Down syndrome (DS) and the leading cause of dependence in people with DS. The neuronal mechanisms underlying DS‐associated cognitive impairment remain unclear, hampering the development of effective therapeutics. Protein synthesis (mRNA translation) deficits associated with aberrant phosphorylation of the eukaryotic elongation factor 2 (eEF2) by its kinase eEF2K are implicated in dementia syndromes. It is unclear whether and how eEF2 phosphorylation and the eEF2K signaling regulation are involved in DS pathogenesis. The relevant references are cited.
2. **Interpretation**: We have shown in the current study that eEF2 phosphorylation was abnormally elevated in the brain of patients with DS and DS mouse models. Inhibition of eEF2 phosphorylation through genetic or pharmacological approaches improved multiple aspects of DS‐associated pathophysiology including protein synthesis deficiency, synaptic failure, and cognitive impairments. Our findings support the hypothesis that eEF2K signaling dysregulation and related mRNA translation deficits play a crucial role in synaptic and cognitive impairments associated with DS.
3. **Future directions**: Future studies in more clinically relevant settings if applicable (e.g., clinical trial) are desired to further determine whether targeting eEF2K signaling could be a feasible therapeutic strategy for cognitive deficits in patients with DS. It is also critical to develop novel and effective small‐molecule eEF2K inhibitors.

## 2 METHODS

### 2.1 Study design

We used a power analysis to calculate the sample size necessary to achieve a reliable measurement of the effect, and we were able to use the appropriate number of animals according to the preliminary power analysis. We used a Grubbs test to identify outliers in all data sets, and if outliers were identified, those data points were excluded. Our hypothesis was that suppression of the eEF2 phosphorylation either genetically or pharmacologically would alleviate cognitive and synaptic deficits in DS model mice, and that protein synthesis capacity would be restored. The research subjects were animals, specifically Ts65Dn and Dp(16)1Yey DS model mice. This study was a controlled laboratory experiment, and mice were randomly assigned to their treatment group. The study was blinded, and the experimenters did not know the allocated condition of the animals when performing behavioral experiments or analyzing tissue.

### 2.2 *Post mortem* human brain tissues

*Post mortem* human brain tissues including hippocampi, prefrontal cortexes (PFCs), and cerebellums were obtained from the University of Washington School of Medicine Brain Bank. Controls (*n* = 5) were age matched and died of non‐neurological diseases. Patients with DS (*n* = 5) were diagnosed based on chromosomal karyotype and clinical symptoms. Pathological examinations of the brain were further conducted to confirm the diagnosis. Detailed demographic information can be found in Table S1 in supporting information.

### 2.3 Mice

All mice were housed at the Wake Forest University School of Medicine barrier facility under the supervision of the Animal Research Program. Mice adhered to a 12‐hour light/12‐hour dark cycle, with regular feeding, cage cleaning, and 24‐hour food and water access. Both male and female mice were included for experimentation. Ts65Dn breeders were purchased from Jackson Lab (JAX stock # 005252), and the strain was maintained by crossing Ts65Dn heterozygous female (male are sterile) with B6EiC3Sn.BLiAF1/J wild‐type (WT) male (JAX stock # 003647). Dp(16)1Yey (Dp16) breeders were purchased from Jackson Lab (JAX stock # 013530), and the strain was maintained by crossing Dp16 heterozygous males with C57BL/6J WT females (JAX stock # 000664). Homozygous eEF2K^−/−^ mice were generously provided by Dr. Alexey G. Ryazanov from Rutgers, and the strain was on B6 background. For Ts65Dn/eEF2K breeding, eEF2K^−/−^ female mice were first crossed with B6EiC3Sn.BLiAF1/J WT male mice to generate eEF2K^+/−^ mice, then eEF2K^+/−^ male mice were crossed with Ts65Dn female mice to generate littermates with equal ratio of four genotypes (WT, TS65Dn, eEF2K^+/−^, and Ts65Dn/eEF2K^+/−^). The age of Ts65Dn cohorts used in this study were 9 to 12 months. In total 204 mice were used in this cohort, including 99 male and 105 female mice. Similarly, for Dp16/eEF2K breeding, eEF2K^−/−^ male mice were first crossed with C57BL/6J WT female mice to generate eEF2K^+/−^ mice, then Dp16 male mice were crossed with eEF2K^+/−^ female mice to generate littermates with equal ratio of four genotypes (WT, Dp16, eEF2K^+/−^, and Dp16/eEF2K^+/−^). The age of the Dp16 cohorts used in this study were 6 to 9 months. In total 137 mice were used in this cohort, including 74 male and 63 female mice. Genotypes of these mice were verified by polymerase chain reaction. All animal experiments were performed in accordance with the approval of the Institutional Animal Care and Use Committee at Wake Forest University (protocol number A21‐112).

### 2.4 Drug pellet

A‐484954 (Millipore Sigma, catalog # 324516) was sent to Innovative Research of America (Sarasota, Florida), where pellets were manufactured. Pellets were stored at room temperature. Each pellet contained 2.625 mg of either A‐484954 or vehicle, a dose previously established to induce effects on eEF2K in mice.^27^ The pellet could release the drug smoothly over 30 days. Mouse was anesthetized using isoflurane. Once the mouse was adequately sedated, a pellet containing either A‐484954 or vehicle was placed into a 10‐gauge trochar. The skin was pierced with the trochar and the pellet was placed subcutaneously. Antibiotic ointment was applied to the injection site after pellet placement to avoid infection. No postoperative analgesics were administered, as the injection site was relatively small, and mouse did not show signs of pain or distress after pellet placement. After placement, the mouse was monitored for negative side effects of the drug and to ensure lack of injury from surgery.

### 2.5 Electrophysiology

Slices were maintained in artificial cerebrospinal fluid (ACSF) bubbled with 95% O_2_/5% CO_2_ at 32°C. Monophasic, constant‐current stimuli (100 µs) were delivered with a bipolar silver electrode placed in the stratum radiatum of area CA3. Field excitatory postsynaptic potentials (fEPSPs) were recorded using a glass microelectrode from the stratum radiatum of area CA1. The input–output relationship was determined by increasing the magnitudes of stimuli from 0 to 10 mV at a step of 0.5 mV. Paired‐pulse ratio was measured by delivering two identical stimuli separated by 25 to 200 ms at a step of 25 ms. Long‐term potentiation (LTP) was induced using high‐frequency stimulation (HFS) consisting of two 1‐second 100 Hz trains separated by 60 seconds, each delivered at 60% to 70% of the intensity that evoked spiked fEPSPs.

### 2.6 Drug treatments

Drugs were prepared as stock solutions in either dimethyl sulfoxide or distilled water and diluted into ACSF to a final concentration before experiments. For NH125 treatment, slices were incubated at 32°C in a recording chamber containing ACSF saturated with bubbling 95% O_2_ and 5% CO_2_. For nelfinavir treatment, slices were incubated at 32°C for 2 hours in a submersion maintenance chamber containing modified ACSF containing the following: 148 mM NaCl, 3.0 mM KCl, 1.4 mM CaCl_2_, 0.8 mM MgCl_2_, 0.8 mM Na_2_HPO_4_, 0.2 mM NaH_2_PO_4_, and 15 mM glucose, bubbled with 100% O_2_. The final concentration was as follows: NH125 (1 µM, Millipore, catalog # 324515) and nelfinavir (50 µM, AmBeed, catalog # A128893).

### 2.7 Statistics and bioinformatic analysis

Data are presented as mean ± standard error of the mean. Summary data are presented as group means with standard error bars. For comparisons between two groups, a two‐tailed independent Student *t* test was performed. For comparisons among more than two groups, one‐way analysis of variance (ANOVA) was used with Tukey post hoc tests for multiple comparisons. If two levels of factors were involved in multiple group comparisons, two‐way ANOVA was used with Tukey post hoc tests for multiple comparisons. *P* < 0.05 was considered statistically significant. Outliers were determined by Grubbs test. Statistics were performed using Prism 7 software (GraphPad). For proteomic analysis, Gene Ontology (GO) analysis was performed with clusterProfiler package (version 4.2.2) in R (version 4.1.2). Venn diagrams were plotted by Venn Diagram package (version 1.7.3), all other graphs were plotted by ggplot2 package (version 3.3.5) in R (version 4.1.2).

### 2.8 Study approval

All protocols involving animals were approved by the Institutional Animal Care and Use Committee of Wake Forest University School of Medicine. Mice were kept in compliance with the National Institutes of Health (NIH) Guide for the Care and Use of Laboratory Animals. Samples of human tissue were collected in accordance with approved institutional review board protocols. All patients gave informed consent.

## 3 RESULTS

### 3.1 Levels of eEF2 phosphorylation are elevated in the brain of patients with DS and mouse models

**FIGURE 1:**
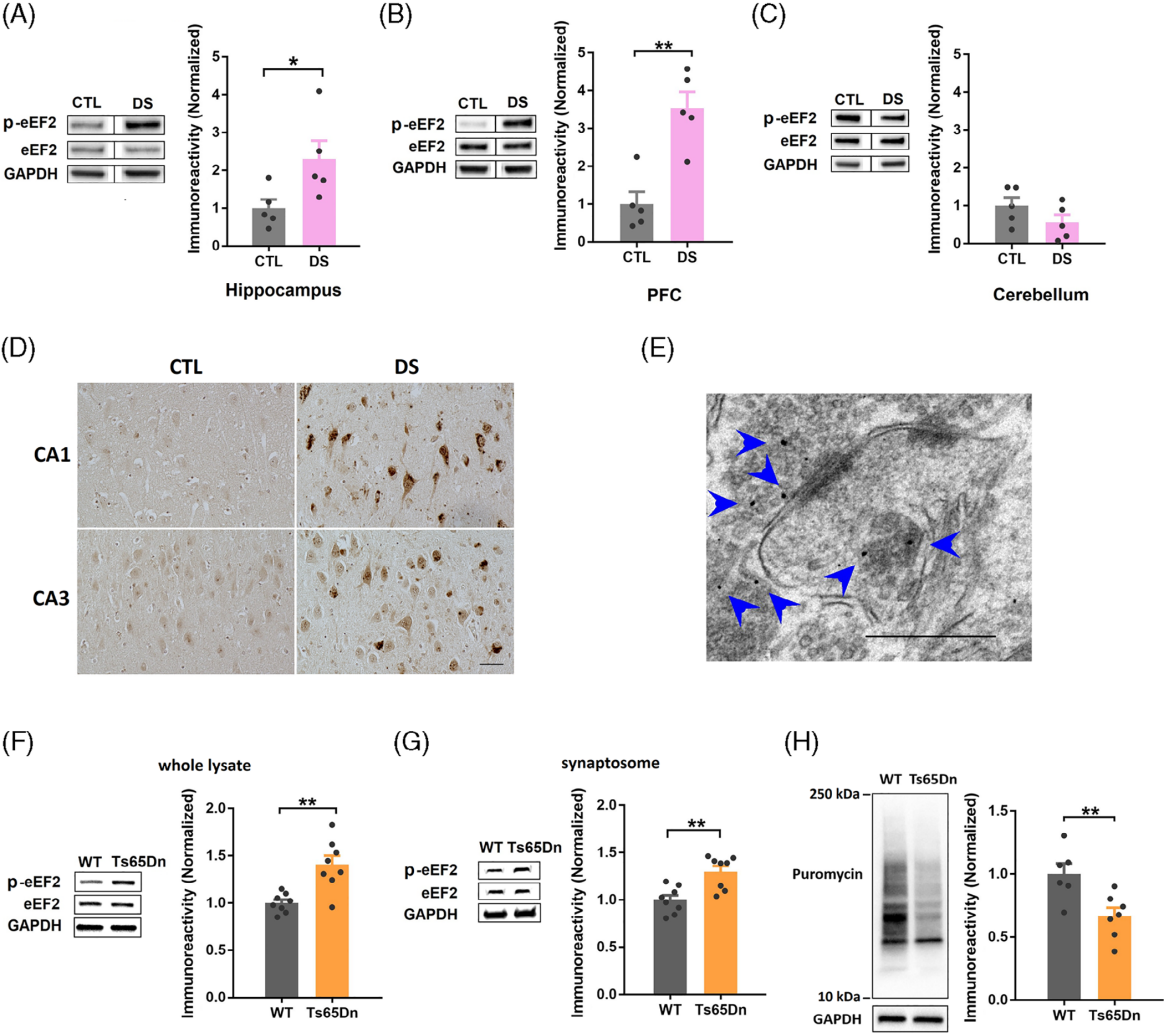
eEF2K phosphorylation is elevated in the brain of patients with DS and Ts65Dn mouse model. A, Representative western blot images of p‐eEF2, eEF2, and GAPDH in *post mortem* hippocampal tissues from CTL and patients with DS, and quantification of p‐eEF2 levels in the two groups. *n* = 5 in each group. * *P *< 0.05, t = 2.395, df = 8, unpaired *t* test. B, Representative western blot images of p‐eEF2, eEF2, and GAPDH in *post mortem* PFC tissues from control and patients with DS, and quantification of p‐eEF2 levels in the two groups. *n* = 5 in each group. ** *P* < 0.01, t = 4.694, df = 8, unpaired *t* test. C, Representative western blot images of p‐eEF2, eEF2, and GAPDH in *post mortem* cerebellum tissues from control and patients with DS, and quantification of p‐eEF2 levels in the two groups. *n* = 5 in each group. t = 1.469, df = 8, *P* = 0.1800, unpaired *t* test. D, Representative IHC images of p‐eEF2 in CA1 and CA3 areas of *post mortem* hippocampal slices from CTL and patients with DS. Scale bar = 50 µm. The experiments were replicated three times. E, Representative immuno‐EM image from the CA1 area of hippocampus of a WT mouse. Gold particles linked to p‐eEF2 could be found both pre‐ and postsynaptic compartments (indicated by blue arrows). Scale bar = 500 nm. F, Representative western blot images of p‐eEF2, eEF2, and GAPDH in hippocampal whole lysates from WT and Ts65Dn mice, and quantification of p‐eEF2 levels in the two groups. *n* = 8 in each group. ** *P* < 0.01, t = 3.767, df = 14, unpaired *t* test. G, Representative western blot images of p‐eEF2, eEF2, and GAPDH in hippocampal synaptosomes from WT and Ts65Dn mice, and quantification of p‐eEF2 levels in the two groups. *n* = 8 in each group. ** *P* < 0.01, t = 4.089, df = 14, unpaired *t* test. H, Representative images and quantification of SUnSET *de novo* protein synthesis assay in hippocampal slices from WT and Ts65Dn mice. WT *n* = 6; Ts65Dn *n* = 7. ** *P* < 0.01, t = 3.161, df = 11, unpaired *t* test. CTL, control; DS, Down syndrome; eEF2, eukaryotic elongation factor 2; eEF2K, eukaryotic elongation factor 2 kinase; EM, electron microscopy; GAPDH, glyceraldehyde 3‐phosphate dehydrogenase; IHC, immunohistochemical; PFC, prefrontal cortex; SUnSET, surface sensing of translation; WT wild type

To investigate whether the eEF2K signaling is dysregulated in the brain of DS, we first assessed eEF2 phosphorylation (at the Thr^56^ site) as a readout of eEF2K activity in *post mortem* brain tissue of patients with DS and age‐matched controls (provided by the NIH Neurobiobank) using Western blot. Demographic information of the subjects is included in the Table S1. Levels of p‐eEF2 were significantly increased in the hippocampus and PFC of patients with DS compared to the controls (Figure 1A and 1B). In comparison, levels of eEF2 phosphorylation in the cerebellum tissue were unaltered between DS and control subjects (Figure 1C). We next performed immunohistochemical experiments to investigate the cellular localization of the eEF2 phosphorylation. We found increased p‐eEF2 staining in the neurons (both soma and neurites) of hippocampal area CA1 and CA3 from patients with DS compared to controls (Figure 1D). To further understand the subcellular localization of the p‐eEF2 signal, we carried out immuno‐electron microscopy experiments in hippocampus from WT mice and revealed that p‐eEF2 was present both in the presynaptic and postsynaptic compartments (Figure 1E). Next, we examined eEF2K signaling regulation in the brain of an established mouse model of DS, the Ts65Dn mouse, which has three copies of most of the genes on the chromosome 16 that are homologues of human chromosome 21 genes.^28^ Consistent with the human data, levels of p‐eEF2 were significantly increased in the hippocampi (both whole lysate and isolated synaptosome) of aged Ts65Dn mice (9–12 months) compared to WT mice (Figure 1F and 1G). We also examined eEF2 phosphorylation in the brain tissue from young (3–4 months) mice and did not observe altered p‐eEF2 levels between WT and DS model mice (data not shown). Because increased eEF2 phosphorylation is linked to inhibition of overall mRNA translation, we assessed general *de novo* protein synthesis in Ts65Dn mice by the surface sensing of translation (SUnSET) assay.^29^ *De novo* protein synthesis, as assessed by puromycin incorporation, was significantly decreased in the hippocampi of Ts65Dn mice compared to littermate WT mice (Figure 1H). Moreover, we validated these findings in a different mouse model of DS, the Dp16 mice.^30^ Consistent with the experimental results from the Ts65Dn mice, levels of p‐eEF2 were significantly increased in the hippocampi (whole lysate and synaptosome) of Dp16 mice (Figure S1A and S1B in supporting information). Overall, *de novo* protein synthesis in the hippocampus was impaired as well in the Dp16 mice compared to WT littermate, as revealed by the SUnSET assay (Figure S1C). In brief, eEF2 phosphorylation was abnormally increased in the DS brain, resulting in impaired translational capacity that may affect memory formation and long‐term synaptic plasticity.^14^, ^31^, ^32^

### 3.2 Suppression of eEF2K with a genetic approach alleviates cognitive deficits in Ts65Dn mice

**FIGURE 2:**
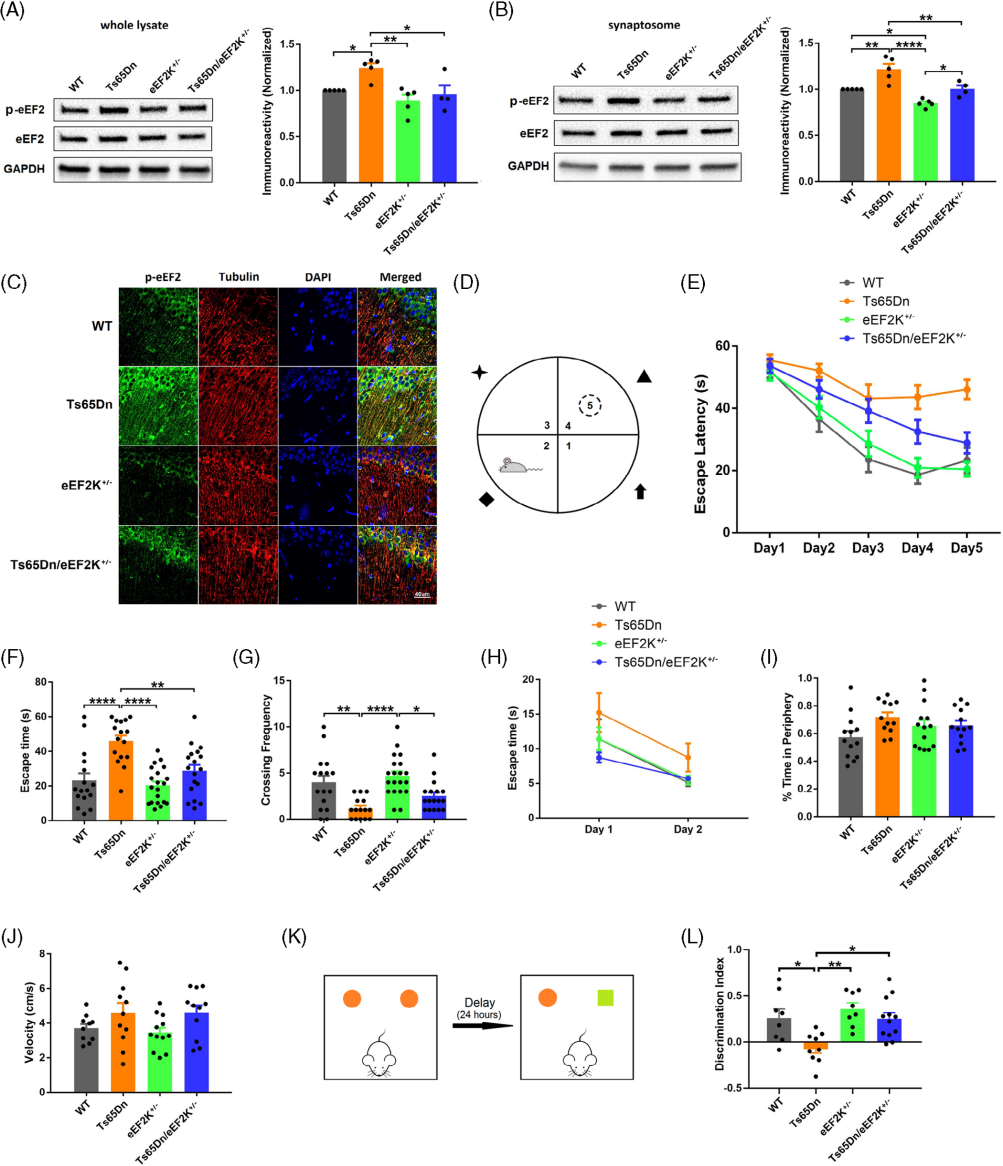
Suppression of eEF2K can restore eEF2 phosphorylation and alleviate cognitive deficits in Ts65Dn mice. A, Representative western blot images of p‐eEF2, eEF2, and GAPDH in hippocampal whole lysates from WT, Ts65Dn, eEF2K^+/−^, and Ts65Dn/eEF2K^+/−^ mice, and quantification of p‐eEF2 levels in the four genotypes. WT *n* = 5; Ts65Dn *n* = 5; eEF2K^+/−^ *n* = 5, Ts65Dn/eEF2K^+/−^ *n* = 4. * *P* < 0.05, ** *P* < 0.01, F(3, 15) = 7.331, one‐way ANOVA with Tukey post hoc test. B, Representative western blot images of p‐eEF2, eEF2, and GAPDH in hippocampal synaptosomes from WT, Ts65Dn, eEF2K^+/−^, and Ts65Dn/eEF2K^+/−^ mice, and quantification of p‐eEF2 levels in the four genotypes. WT *n* = 5; Ts65Dn *n* = 5; eEF2K^+/−^ *n* = 5, Ts65Dn/eEF2K^+/−^ *n* = 4. * *P* < 0.05, ** *P* < 0.01, **** *P* < 0.001, F(3, 15) = 18.01, one‐way ANOVA with Tukey post hoc test. C, Representative immunofluorescence images of p‐eEF2 (green), Tubulin (red), and DAPI (blue) in the CA1 areas of hippocampal slices from WT, Ts65Dn, eEF2K^+/−^, and Ts65Dn/eEF2K^+/−^ mice. Scale bar = 40 µm. The experiments were replicated three times. D, Paradigm of MWM test. E, Escape latencies of WT, Ts65Dn, eEF2K^+/−^, and Ts65Dn/eEF2K^+/−^ mice during 5 day training phase of MWM test. WT *n* = 16; Ts65Dn *n* = 16; eEF2K^+/−^ *n* = 20; Ts65Dn/eEF2K^+/−^ *n* = 18. F, Escape latencies on day 5 of MWM training phase. WT *n* = 16; Ts65Dn *n* = 16; eEF2K^+/−^ *n* = 20; Ts65Dn/eEF2K^+/−^ *n* = 18. ** *P* < 0.01, **** *P* < 0.0001, F(3, 66) = 12.37, one‐way ANOVA with Tukey post hoc test. G, Frequencies of crossing the “platform” of WT, Ts65Dn, eEF2K^+/−^, and Ts65Dn/eEF2K^+/−^ mice in the probe trial. WT *n* = 16; Ts65Dn *n* = 15; eEF2K^+/−^ *n* = 20; Ts65Dn/eEF2K^+/−^ *n* = 17. * *P* < 0.05, ** *P* < 0.01, **** *P* < 0.0001, F(3, 64) = 9.202, one‐way ANOVA with Tukey post hoc test. H, Escape latencies of WT, Ts65Dn, eEF2K^+/−^, and Ts65Dn/eEF2K^+/−^ mice in the VP test. WT *n* = 14; Ts65Dn *n* = 13; eEF2K^+/−^ *n* = 17; Ts65Dn/eEF2K^+/−^ *n* = 16. I, Ratio of time spent in the periphery in OF test of WT, Ts65Dn, eEF2K^+/−^, and Ts65Dn/eEF2K^+/−^ mice. WT *n* = 13; Ts65Dn *n* = 12; eEF2K^+/−^ *n* = 14; Ts65Dn/eEF2K^+/−^ *n* = 13. F(3, 48) = 2.150, *P* = 0.1063, one‐way ANOVA with Tukey post hoc test. J, Velocities of WT, Ts65Dn, eEF2K^+/−^, and Ts65Dn/eEF2K^+/−^ mice in the OF test. WT *n* = 10; Ts65Dn *n* = 11; eEF2K^+/−^ *n *= 12; Ts65Dn/eEF2K^+/−^ *n* = 11. F(3, 40) = 2.238, *p *= 0.0987, one‐way ANOVA with Tukey post hoc test. K, Paradigm of NOR test. L, Discrimination index of WT, Ts65Dn, eEF2K^+/−^, and Ts65Dn/eEF2K^+/−^ mice in the NOR test. WT *n* = 8; Ts65Dn *n* = 9; eEF2K^+/−^ *n* = 8; Ts65Dn/eEF2K^+/−^ *n* = 12. * *P* < 0.05, ** *P* < 0.01, F(3, 33) = 6.505, one‐way ANOVA with Tukey post hoc test. ANOVA, analysis of variance; DS, Down syndrome; eEF2, eukaryotic elongation factor 2; eEF2K, eukaryotic elongation factor 2 kinase; GAPDH, glyceraldehyde 3‐phosphate dehydrogenase; MWM, Morris water maze; NOR, novel object recognition; OF, open field; VP, visible platform; WT wild type

To further investigate the association between eEF2 hyperphosphorylation and DS pathophysiology, we crossed male eEF2K heterozygous knockout mice (eEF2K^+/−^)^25^ with female Ts65Dn mice to generate the Ts65Dn/eEF2K^+/−^ double mutant mice, along with three other experimental groups: WT, Ts65Dn, and eEF2K^+/−^. Western blot experiments showed that elevated levels of p‐eEF2 in TS65Dn mice were restored in Ts65Dn/eEF2K^+/−^ mice, either in the whole lysate or the synaptosome of hippocampi, to the level comparable to WT mice (Figure 2A and 2B). Additionally, suppression of eEF2K did not affect total eEF2 protein levels across the four genotypes (Figure 2A and 2B). Immunofluorescence staining revealed hyperphosphorylation of eEF2 (green) in both the soma and dendrites of pyramidal neurons in hippocampi of Ts65Dn mice compared to WT mice, and suppression of eEF2K was able to decrease eEF2 phosphorylation in hippocampal neurons in Ts65Dn mice (Figure 2C). Next, we conducted a series of behavioral tests to assess cognitive function of these mice at the age of 9 to 12 months old. In the Morris water maze (MWM) test, which is known for evaluation of spatial learning and memory, we trained mice to find a hidden platform according to spatial cues around a water tank (Figure 2D). Compared to WT mice, the Ts65Dn DS mice displayed impaired learning and memory as indicated by longer day‐to‐day escape latency (time to locate the hidden platform) during the training phase, and less “platform” crossing during the probe trial (Figure 2E‐G). In contrast, suppression of eEF2K improved DS‐associated spatial learning and memory deficits, as indicated by normal (indistinguishable from WT mice) performance of Ts65Dn/eEF2K^+/−^ mice (Figure 2E‐G). In addition, eEF2K^+/−^ mice showed normal learning and memory during the MWM test (Figure 2E‐G). To exclude memory‐independent effects associated with eEF2K suppression such as vision and swimming ability, we also conducted visible platform (VP) test. There were no significant differences across the four genotype mice in day 2 of the VP test, and all four genotype mice showed improved performance in day 2 compared to day 1 (Figure 2H). Next, we conducted open field (OF) test to assess locomotor activity and anxiety phenotype of the mice.^33^ We did not observe significant differences across all four groups of mice during the OF test in the evaluation of locomotor activity (moving speed and distance) and anxiety (ratio of time spent in the periphery; Figure 2I and 2J). We further performed the novel object recognition (NOR) test to assess long‐term recognition memory in these mice.^34^ As demonstrated in the discrimination index ([time spent with novel object – familiar object] / total time) data, Ts65Dn mice, compared to WT mice, were unable to distinguish novel and familiar objects, indicating long‐term recognition memory deficit (Figure 2K and 2L). Importantly, suppression of eEF2K alleviated such cognitive deficits in Ts65Dn mice, as indicated by significantly improved discrimination index of the Ts65Dn/eEF2K^+/−^ mice (Figure 2L). In summary, suppression of eEF2K can restore eEF2 phosphorylation levels and alleviate cognitive deficits in the Ts65Dn DS mouse model.

### 3.3 Suppression of eEF2K with a genetic approach can ameliorate defects of dendritic spine morphology and long‐term synaptic plasticity in Ts65Dn mice

**FIGURE 3:**
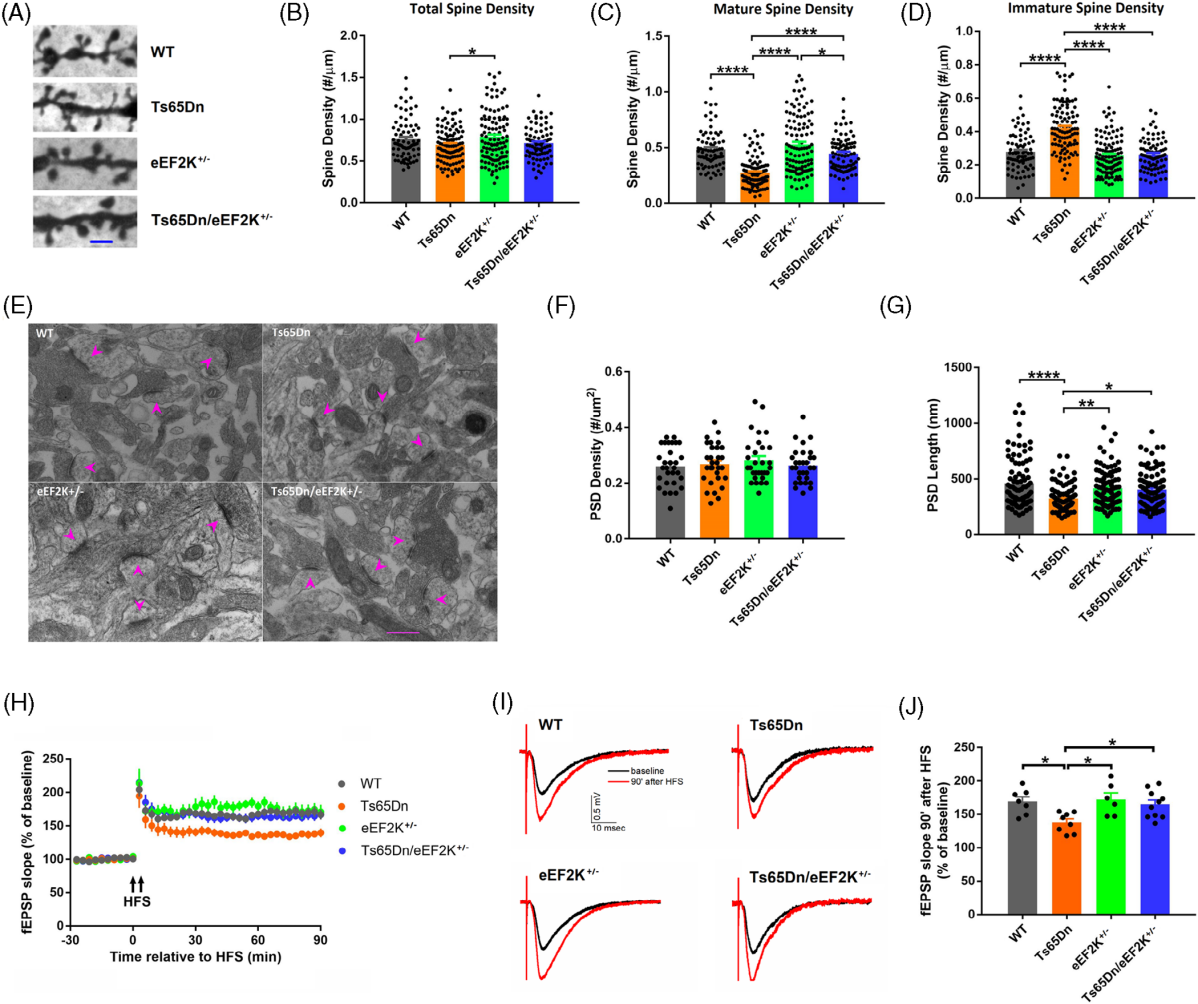
Suppression of eEF2K can ameliorate defects of dendritic spine morphology and long‐term synaptic plasticity in Ts65Dn mice. A, Representative Golgi staining images in the CA1 areas of hippocampi from WT, Ts65Dn, eEF2K^+/−^, and Ts65Dn/eEF2K^+/−^ mice. Scale bar = 2 µm. B, Cumulative data of total spine densities of hippocampal CA1 dendrites from WT, Ts65Dn, eEF2K^+/−^, and Ts65Dn/eEF2K^+/−^ mice. WT *n* = 77 dendrites; Ts65Dn *n* = 100 dendrites; eEF2K^+/−^ *n* = 109 dendrites; Ts65Dn/eEF2K^+/−^ *n* = 83 dendrites. Three mice in each group. * *P* < 0.05, F(3, 365) = 3.210, one‐way ANOVA with Tukey post hoc test. C, Cumulative data of mature spine densities of hippocampal CA1 dendrites from WT, Ts65Dn, eEF2K^+/−^, and Ts65Dn/eEF2K^+/−^ mice. WT *n* = 77 dendrites; Ts65Dn *n* = 100 dendrites; eEF2K^+/−^ *n* = 109 dendrites; Ts65Dn/eEF2K^+/−^ *n *= 83 dendrites. Three mice in each group. * *P* < 0.05, **** *P* < 0.0001, F(3, 365) = 35.45, one‐way ANOVA with Tukey post hoc test. D, Cumulative data of immature spine densities of hippocampal CA1 dendrites from WT, Ts65Dn, eEF2K^+/−^, and Ts65Dn/eEF2K^+/−^ mice. WT *n* = 77 dendrites; Ts65Dn *n* = 100 dendrites; eEF2K^+/−^ *n* = 108 dendrites; Ts65Dn/eEF2K^+/‐^ *n* = 82 dendrites. Three mice in each group. **** *P* < 0.0001, F(3, 365) = 43.53, one‐way ANOVA with Tukey post hoc test. E, Representative EM images in the CA1 areas of hippocampi from WT, Ts65Dn, eEF2K^+/−^, and Ts65Dn/eEF2K^+/−^ mice. Scale bar = 500 nm. F, Cumulative data of PSD densities in the CA1 areas of hippocampi from WT, Ts65Dn, eEF2K^+/−^, and Ts65Dn/eEF2K^+/−^ mice. *n* = 30 images from three mice in each group. F(3, 116) = 0.5608, *P* = 0.6419, one‐way ANOVA with Tukey post hoc test. G, Cumulative data of PSD lengths in the CA1 areas of hippocampi from WT, Ts65Dn, eEF2K^+/−^, and Ts65Dn/eEF2K^+/−^ mice. WT *n* = 90 synapses; Ts65Dn *n* = 92 synapses; eEF2K^+/−^ *n* = 96 synapses; Ts65Dn/eEF2K^+/−^ *n* = 94 synapses. Three mice in each group. * *P* < 0.05, ** *P* < 0.01, ****P < 0.0001, F(3, 368) = 9.876, one‐way ANOVA with Tukey post hoc test. H, Hippocampal LTP in WT, Ts65Dn, eEF2K^+/−^, and Ts65Dn/eEF2K^+/−^ mice. Arrows indicate HFS. WT *n* = 7; Ts65Dn *n* = 8; eEF2K^+/−^ *n* = 6; Ts65Dn/eEF2K^+/−^ *n* = 10. I, Representative fEPSP traces before and after HFS in WT, Ts65Dn, eEF2K^+/−^, and Ts65Dn/eEF2K^+/−^ mice. J, Cumulative data showing fEPSP slopes at 90 minutes after HFS in WT, Ts65Dn, eEF2K^+/−^, and Ts65Dn/eEF2K^+/−^ mice. WT *n* = 7; Ts65Dn *n* = 8; eEF2K^+/−^ *n* = 6; Ts65Dn/eEF2K^+/−^ *n *= 10. * *P* < 0.05, F(3, 27) = 4.817, one‐way ANOVA with Tukey post hoc test. ANOVA, analysis of variance; eEF2, eukaryotic elongation factor 2; eEF2K, eukaryotic elongation factor 2 kinase; EM, electron microscopy; fEPSP, field excitatory postsynaptic potential; GAPDH, glyceraldehyde 3‐phosphate dehydrogenase; HFS, high‐frequency stimulation; PSD, postsynaptic density; WT wild type

Spine morphology is a critical indicator of synaptic integrity and is associated with memory formation and synaptic plasticity.^35^, ^36^ Patients with DS and mouse models are characterized by synaptic abnormalities, including decreased mature spine density, and increased immature spine density in the cerebral cortex and hippocampus.^37^ We used a rapid Golgi staining protocol^38^to assess morphological changes of the dendritic spines in the hippocampus (Figure 3A). Analysis of spine density and subtypes was based on published guidelines.^39^, ^40^ There was no significant difference in total spine density between WT and Ts65Dn mice, which was consistent with previous reports.^37^, ^41^ Interestingly, total spine density was increased in eEF2K^+/−^ mice compared to Ts65Dn mice (Figure 3B). Suppression of eEF2K did not alter total spine density count in Ts65Dn mice (Figure 3B). Further analysis on mature (mushroom, stubby, and branched) and immature (filopodia and thin) spines revealed that mature spine density was significantly decreased while immature spine density was significantly increased in the hippocampus of Ts65Dn mice compared to WT mice (Figure 3C and 3D). Importantly, suppression of eEF2K was able to restore such spine abnormality (reduced mature spine and increased immature spine density) in Ts65Dn mice (Figure 3C and 3D). Next, we applied the transmission electron microscopy (TEM) method to evaluate synaptic changes in the hippocampus (Figure 3E). Densities of postsynaptic density (PSD), which was a distinct structure of synapse, were not significantly altered across the four genotypes (Figure 3F). Meanwhile, the size of PSD, which was measured by PSD length, was significantly decreased in Ts65Dn mice compared to WT mice (Figure 3G). Moreover, suppression of eEF2K improved such defects in PSD in Ts65Dn mice (Figure 3G). Further, we performed synaptic electrophysiology experiments to assess long‐term synaptic plasticity in acute hippocampal slices derived from the mice of the four genotypes. First, we measured the input/output (I/O) relationship and paired‐pulse facilitation (PPF) and did not find significant differences across the four genotypes, suggesting unaltered basal synaptic transmission and presynaptic function with eEF2K regulation (Figure S2A and S2B in supporting information). We next examined protein synthesis–dependent LTP (induced by high‐frequency stimulus), a major form of synaptic plasticity that is considered a cellular model for learning and memory.^42^ Hippocampal LTP was impaired in Ts65Dn mice compared to WT mice, which is consistent with previous studies.^37^, ^43^ Notably, suppression of eEF2K alleviated LTP impairment in Ts65Dn mice (Figure 3H‐J). In conclusion, suppression of eEF2K can alleviate morphological defects in dendritic spine and synapse as well as long‐term synaptic plasticity impairment in Ts65Dn mice.

### 3.4 Repression of eEF2K restores multifaceted abnormalities in Dp16 DS model mice

**FIGURE 4:**
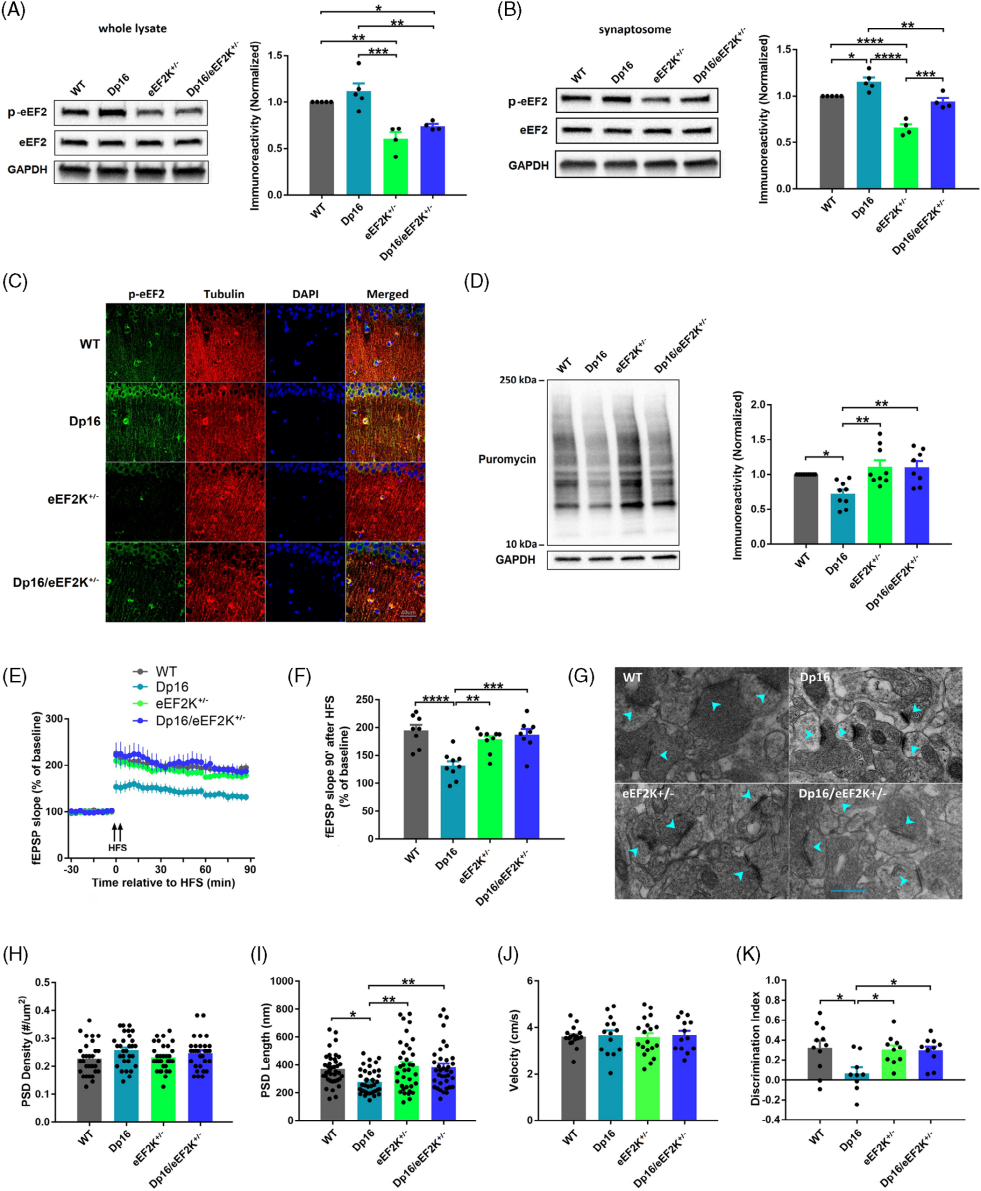
Suppression of eEF2K restores multifaceted abnormalities in Dp16 mice. A, Representative western blot images of p‐eEF2, eEF2, and GAPDH in hippocampal whole lysates from WT, Dp16, eEF2K^+/−^, and Dp16/eEF2K^+/−^ mice, and quantification of p‐eEF2 levels in the four genotypes. WT *n* = 5; Dp16 *n* = 5; eEF2K^+/−^ *n* = 4, Dp16/eEF2K^+/−^ *n* = 4. * *P* < 0.05, ** *P* < 0.01, *** *P* < 0.001, F(3, 14) = 15.91, one‐way ANOVA with Tukey post hoc test. B, Representative western blot images of p‐eEF2, eEF2, and GAPDH in hippocampal synaptosomes from WT, Dp16, eEF2K^+/−^, and Dp16/eEF2K^+/−^ mice, and quantification of p‐eEF2 levels in the four genotypes. WT *n *= 5; Dp16 *n* = 5; eEF2K^+/−^ *n* = 4, Dp16/eEF2K^+/−^ *n* = 4. * *P* < 0.05, ** *P* < 0.01, *** *P* < 0.001, **** *P* < 0.0001, F(3, 14) = 33.97, one‐way ANOVA with Tukey post hoc test. C, Representative immunofluorescence images of p‐eEF2 (green), Tubulin (red), and DAPI (blue) in the CA1 areas of hippocampal slices from WT, DP16, eEF2K^+/−^, and Dp16/eEF2K^+/−^ mice. Scale bar = 40 µm. The experiments were replicated 3 times. D, Representative images and quantification of the SUnSET assay in hippocampal slices from WT, Dp16, eEF2K^+/−^, and Dp16/eEF2K^+/−^ mice, and quantification of puromycin levels in the four genotypes. WT *n* = 9; Dp16 *n* = 9; eEF2K^+/−^ *n* = 9, Dp16/eEF2K^+/−^ *n* = 8. * *P* < 0.05, ** *P* < 0.01, F(3, 31) = 7.120, one‐way ANOVA with Tukey post hoc test. E, Hippocampal LTP in WT, Dp16, eEF2K^+/−^, and Dp16/eEF2K^+/−^ mice. Arrows indicate HFS. WT *n* = 8; Dp16 *n* = 9; eEF2K^+/−^ *n* = 10; Dp16/eEF2K^+/−^ *n* = 8. F, Cumulative data of fEPSP slopes at 90 minutes after HFS in WT, Dp16, eEF2K^+/−^, and Dp16/eEF2K^+/−^ mice. WT *n* = 8; Dp16 *n* = 9; eEF2K^+/−^ *n* = 10; Dp16/eEF2K^+/−^ *n* = 8. ** *P* < 0.01, *** *P* < 0.001, **** *P* < 0.0001, F(3, 31) = 11.35, one‐way ANOVA with Tukey post hoc test. G, Representative TEM images in the CA1 areas of hippocampi from WT, Dp16, eEF2K^+/−^, and Dp16/eEF2K^+/−^ mice. Scale bar = 500 nm. H, Cumulative data of PSD densities in the CA1 areas of hippocampi from WT, Dp16, eEF2K^+/−^, and Dp16/eEF2K^+/−^ mice. *n* = 30 images from three mice in each group. F(3, 117) = 1.864, *P* = 0.1395, one‐way ANOVA with Tukey post hoc test. I, Cumulative data of PSD lengths in the CA1 areas of hippocampi from WT, Dp16, eEF2K^+/−^, and Dp16/eEF2K^+/−^ mice. WT *n* = 38 synapses; Dp16 *n* = 41 synapses; eEF2K^+/−^ *n* = 36 synapses; Dp16/eEF2K^+/−^ *n* = 37 synapses. Three mice in each group. * *P* < 0.05, ** *P *< 0.01, F(3, 148) = 5.940, one‐way ANOVA with Tukey post hoc test. J, Velocities of WT, Dp16, eEF2K^+/−^, and Dp16/eEF2K^+/−^ mice in OF test. WT *n* = 17; Dp16 *n* = 14; eEF2K^+/−^ *n* = 19; Dp16/eEF2K^+/−^ *n* = 13. F(3, 59) = 0.062, *P* = 0.9798, one‐way ANOVA with Tukey post hoc test. K, Discrimination index of WT, Dp16, eEF2K^+/−^, and Dp16/eEF2K^+/−^ mice in NOR test. WT *n *= 11; Dp16 *n* = 9; eEF2K^+/−^ *n* = 10; Dp16/eEF2K^+/−^ *n* = 11. * *P* < 0.05, F(3, 37) = 4.319, one‐way ANOVA with Tukey post hoc test. ANOVA, analysis of variance; eEF2, eukaryotic elongation factor 2; eEF2K, eukaryotic elongation factor 2 kinase; fEPSP, field excitatory postsynaptic potential; GAPDH, glyceraldehyde 3‐phosphate dehydrogenase; HFS, high‐frequency stimulation; LTP, long‐term potentiation; NOR, novel object recognition; OF, open field; PSD, postsynaptic density; SUnSET, surface sensing of translation; TEM, transmission electron microscopy; WT wild type

To verify our findings in Ts65Dn mice with eEF2K suppression, we also crossed the eEF2K^+/−^ mice with another established mouse model of DS, the Dp16 mice in which the entire chromosome 16 that is homologous to human chromosome 21 has been triplicated.^30^ The experimental groups include WT, DP16, eEF2^+/−^, and Dp16/eEF2^+/−^. Similar to the findings in Ts65Dn mice, levels of p‐eEF2 were increased either in the whole lysate or synaptosome of hippocampi of Dp16 mice compared to WT mice, which was restored by suppression of eEF2K (Figure 4A and 4B). Immunofluorescence staining of the hippocampal slices showed increased staining of p‐eEF2 level (green) in both the soma and dendrites of hippocampal neurons in Dp16 mice compared to WT mice, which was reduced by eEF2K suppression (Figure 4C). Moreover, deficits of *de novo* protein synthesis (assessed by SUnSET) in the hippocampus of Dp16 mice were alleviated with suppression of eEF2K (Figure 4D). Moreover, eEF2K reduction mitigates hippocampal LTP failure in the Dp16 mice (Figure 4E and 4F), which is consistent with the electrophysiology data from the Ts65Dn cohort. In addition, there were no significant differences in I/O relationship and PPF performance across the four genotypes (Figure S2C and S2D). Analysis of the TEM images from the hippocampal slices revealed that PSD densities were not significantly different across the four genotypes (Figure 4G and 4H). In agreement with the findings from the Ts65Dn experiments, PSD length was significantly decreased in Dp16 mice compared to WT mice, which was improved by eEF2K suppression (Figure 4I). We also examined spine morphology, and found no significant difference in total spine density across the four genotypes. Meanwhile, mature spine density was significantly decreased, and immature spine density was significantly increased in Dp16 mice compared to WT mice, while suppression of eEF2K corrected these defects (Figure S3E‐H in supporting information). Last, we conducted the same battery of behavioral tests to evaluate the cognitive function of the mice. In the OF test, the four genotypes showed similar locomotor activities and anxiety level (Figure 4J, Figure S3A). In the NOR test, deficit in long‐term recognition memory was identified in Dp16 mice compared to WT mice based on the discrimination index, and such deficits were improved by genetic repression of eEF2K (Figure 4K). Further, spatial learning and memory deficits in Dp16 mice, assessed by the MWM test, were also improved in the Dp16/eEF2K^+/−^ mice (Figure S3B and S3C). In the VP test, there was no significant difference across the four genotypes and all the mice showed improvement in performance on Day 2 (Figure S3D). In conclusion, suppression of eEF2K improved multiple pathophysiology in Dp16 mice including protein synthesis deficits, synaptic failure, and cognitive impairments. Such results were consistent with the findings from the Ts65Dn mice cohort.

### 3.5 Effects of eEF2K suppression on protein profiling in Dp16 DS model mice

**FIGURE 5:**
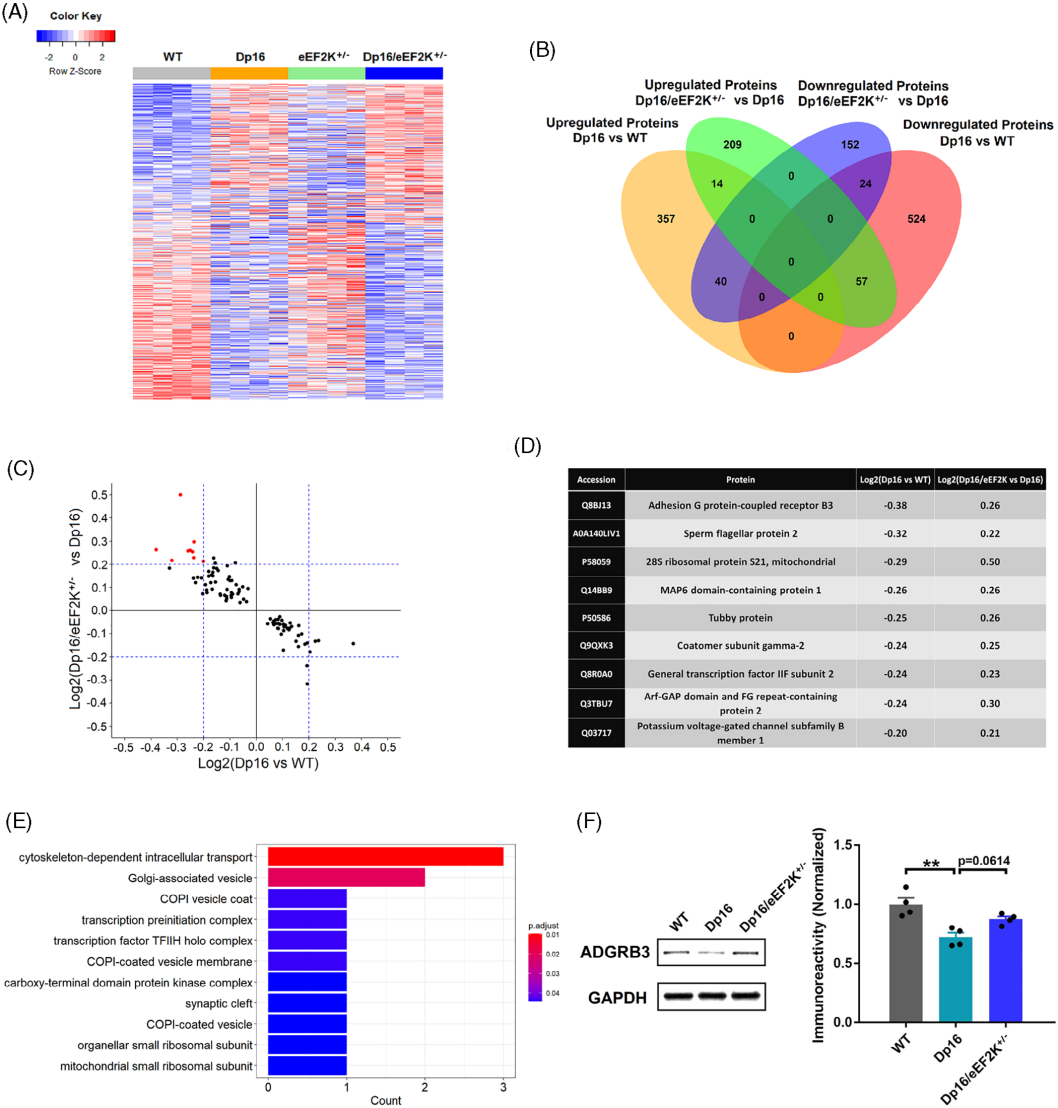
Effects of eEF2K suppression on protein profiling in Dp16 DS model mice. A, Heat map of differentially expressed proteins (*n* = 1328) in WT, Dp16, eEF2K^+/−^, and Dp16/eEF2K^+/−^ mice. n = 4 in each group. One‐way ANOVA. B, Venn diagram of upregulated proteins in Dp16 versus WT (*n* = 411, tan), downregulated proteins in Dp16 versus WT (*n* = 605, pink), upregulated proteins in Dp16/eEF2K^+/−^ versus Dp16 (*n* = 280, green), and downregulated proteins in Dp16/eEF2K^+/−^ versus Dp16 (*n* = 216, purple). Forty proteins were upregulated in Dp16 mice while could be downregulated by eEF2K knockdown, and 57 proteins were downregulated in Dp16 while could be upregulated by eEF2K knockdown. Unpaired *t* test. C, Plot of the 97 (40+57) proteins which were dysregulated in Dp16 while could be corrected by eEF2K knockdown by their fold changes. Increased or decreased by at least 20% in fold changes were considered for further analysis (9 proteins, red). D, Description of the nine proteins identified in (C). E, Gene Ontology analysis of the nine proteins identified. F, Representative western blot images of ADGRB3 and GAPDH in hippocampal tissues from WT, Dp16, and Dp16/eEF2K^+/−^ mice, and quantification of ADGRB3 levels in the three groups. *n* = 4 in each group. ** *P* < 0.01, F(2, 9) = 11.78, one‐way ANOVA with Tukey post hoc test. ADGRB3, adhesion G protein‐coupled receptor B3; ANOVA, analysis of variance; eEF2, eukaryotic elongation factor 2; eEF2K, eukaryotic elongation factor 2 kinase; GAPDH, glyceraldehyde 3‐phosphate dehydrogenase; WT wild type

To investigate the downstream effectors of eEF2K suppression in DS mice, we did proteomic analysis on the hippocampal tissues from WT, Dp16, eEF2K^+/−^, and Dp16/eEF2K^+/−^ mice using tandem mass tag (TMT) mass spectrometry (MS). This method allowed multiplexed analysis of samples at one time and increased sensitivity and reproducibility over the label‐free MS method.^44^ A total of 5338 proteins were identified and quantified after eliminating contaminants and missing values (original proteomic dataset can be found at *MassIVE* with accession number MSV000093209). Among these proteins, 1328 proteins were differentially expressed across the four genotypes. A heat map was generated to demonstrate the protein profile (Figure 5A). We wanted to determine those dysregulated proteins in Dp16 mice (compared to WT mice) that could be corrected by eEF2K suppression. We found 411 upregulated and 605 downregulated proteins in Dp16 mice compared to WT mice. We then identified 280 upregulated and 216 downregulated proteins in Dp16/eEF2K^+/−^ mice compared to Dp16 mice. Comparison between upregulated proteins in Dp16 mice and downregulated proteins in Dp16/eEF2K^+/−^ mice revealed 40 proteins that were shared by the two cohorts. Meanwhile, comparison between downregulated proteins in Dp16 mice and upregulated proteins in Dp16/eEF2K^+/−^ mice identified 57 proteins shared by the two cohorts (Figure 5B). Those were dysregulated proteins in Dp16 mice which could be corrected by eEF2K suppression. We then plotted these proteins with *x* axis as log2 of fold changes of Dp16/WT and *y* axis as log2 of fold changes of Dp16/eEF2K^+/−^/Dp16. We set a threshold of either increased or decreased by at least 20% between groups. The data revealed nine proteins whose expression was decreased in Dp16 mice (compared to WT) and was restored in Dp16/eEF2K^+/−^ mice (Figure 5C and 5D). We then did GO analysis on these proteins and found that they mainly belong to the categories of proteins that are involved in protein synthesis and synaptic functions such as cytoskeleton‐dependent intracellular transport, Golgi‐associated vesicle, COPI‐coated vesicle, synaptic cleft, and ribosomal subunit (Figure 5E). One notable protein was the adhesion G protein‐coupled receptor B3 (ADGRB3), which is involved in synaptogenesis. ^45^ Western blot confirmed that ADGRB3 was decreased in Dp16 mice compared to WT and was improved in Dp16/eEF2K^+/−^ mice (*P* = 0.06; Figure 5F).

### 3.6 Overexpression of PQBP1 alleviates synaptic failure and behavioral deficits in Ts65Dn mice

**FIGURE 6:**
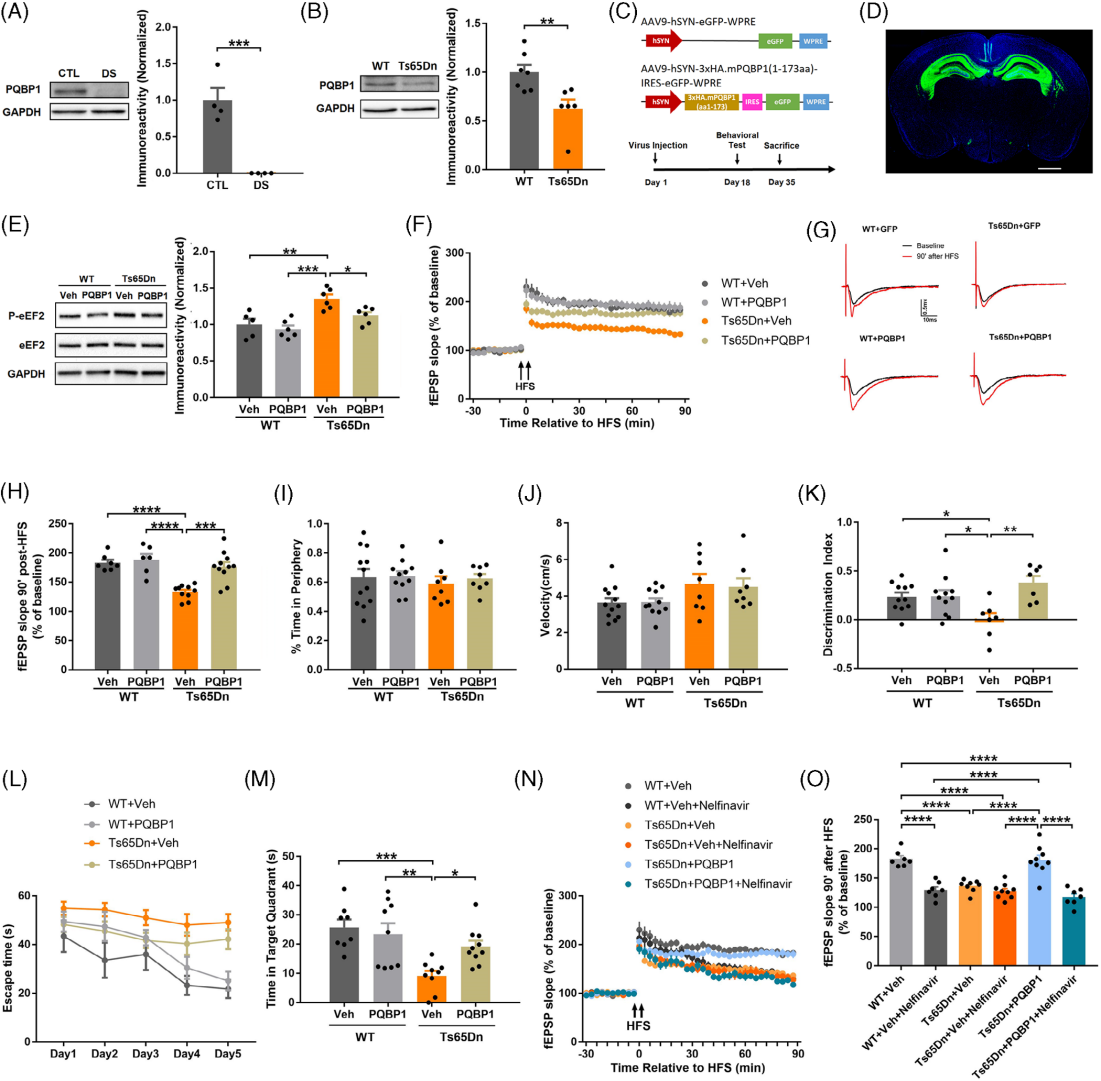
Overexpression of hippocampal PQBP1 alleviates synaptic failure and behavioral deficits in Ts65Dn mice. A, Representative western blot images of PQBP1 and GAPDH in hippocampal tissues from control and patients with DS, and quantification of PQBP1 levels in the two groups. *n* = 4 in each group. *** *P* < 0.001, t = 5.978, df = 6, unpaired *t* test. B, Representative western blot images of PQBP1 and GAPDH in hippocampal tissues from WT and Ts65Dn mice, and quantification of PQBP1 levels in the two groups. WT *n* = 7; Ts65Dn n = 6. ** *P* < 0.01, t = 3.173, df = 11, unpaired *t* test. C, Construction of vehicle AAV9 vector (upper) and PQBP1 AAV9 vector (middle), and timeline of the experiments (bottom). D, Representative immunofluorescence image of GFP (green) and DAPI (blue) from a mouse brain slice injected with the virus. Scale bar = 1000 µm. E, Representative western blot images of p‐eEF2, eEF2, and GAPDH in hippocampal tissues from WT+vehicle, WT+PQBP1, Ts65Dn+vehicle, and Ts65Dn+PQBP1 mice, and quantification of p‐eEF2 levels in the four groups. WT+vehicle *n* = 5; WT+PQBP1 *n* = 6; Ts65Dn+vehicle *n* = 6; Ts65Dn+PQBP1 *n* = 6. * *p* < 0.05, ** *p* < 0.01, *** *p* < 0.001, interaction F(1, 19) = 2.007, virus factor F(1, 19) = 6.430, genotype factor F(1, 19) = 22.06, two‐way ANOVA with Tukey post hoc test. F, Hippocampal LTP in WT+vehicle, WT+PQBP1, Ts65Dn+vehicle, and Ts65Dn+PQBP1 mice. Arrows indicate HFS. WT+vehicle *n* = 7; WT+PQBP1 *n* = 6; Ts65Dn+vehicle *n* = 10; Ts65Dn+PQBP1 *n* = 11. G, Representative traces before and after HFS in WT+vehicle, WT+PQBP1, Ts65Dn+vehicle, and Ts65Dn+PQBP1 mice. H, fEPSP slopes at 90 minutes after HFS in WT+vehicle, WT+PQBP1, Ts65Dn+vehicle, and Ts65Dn+PQBP1 mice. WT+vehicle *n* = 7; WT+PQBP1 *n* = 6; Ts65Dn+vehicle *n* = 10; Ts65Dn+PQBP1 *n* = 11. *** *P* < 0.001, **** *P* < 0.0001, interaction F(1, 30) = 7.401, virus factor F(1, 30) = 11.94, genotype factor F(1, 30) = 19.24, two‐way ANOVA with Tukey post hoc test. I, Ratio of time spent in the periphery in OF test of WT+vehicle, WT+PQBP1, Ts65Dn+vehicle, and Ts65Dn+PQBP1 mice. WT+vehicle *n* = 12; WT+PQBP1 *n* = 11; Ts65Dn+vehicle *n* = 8; Ts65Dn+PQBP1 *n* = 8. Interaction F(1, 35) = 0.0832, *P* = 0.7747; virus factor F(1, 35) = 0.2092, *P* = 0.6502; genotype factor F(1, 35) = 0.3892, *P* = 0.5368; two‐way ANOVA with Tukey post hoc test. J, Velocities of WT+vehicle, WT+PQBP1, Ts65Dn+vehicle, and Ts65Dn+PQBP1 mice in OF test. WT+vehicle *n* = 12; WT+PQBP1 *n* = 11; Ts65Dn+vehicle *n* = 8; Ts65Dn+PQBP1 *n* = 8. Interaction F(1, 35) = 0.0692, *P* = 0.7941; virus factor F(1, 35) = 0.0272, *P* = 0.8700; genotype factor F(1, 35) = 6.731, *P* = 0.0137; two‐way ANOVA with Tukey post hoc test. K, Discrimination index of WT+vehicle, WT+PQBP1, Ts65Dn+vehicle, and Ts65Dn+PQBP1 mice in NOR test. WT+vehicle *n *= 11; WT+PQBP1 *n* = 11; Ts65Dn+vehicle *n* = 7; Ts65Dn+PQBP1 *n* = 7. * *P* < 0.05, ** *P* < 0.01, interaction F(1, 32) = 9.338, virus factor F(1, 32) = 9.894, genotype factor F(1, 32) = 0.6291, two‐way ANOVA with Tukey post hoc test. L, Escape latencies of WT+vehicle, WT+PQBP1, Ts65Dn+vehicle, and Ts65Dn+PQBP1 mice during 5 day training phase of MWM test. WT+vehicle *n* = 8; WT+PQBP1 *n* = 9; Ts65Dn+vehicle *n* = 10; Ts65Dn+PQBP1 *n* = 10. M, Time in the target quadrant of WT+vehicle, WT+PQBP1, Ts65Dn+vehicle, and Ts65Dn+PQBP1 mice in the probe trial. WT+vehicle *n* = 8; WT+PQBP1 *n* = 9; Ts65Dn+vehicle *n* = 9; Ts65Dn+PQBP1 *n* = 10. * *P* < 0.05, ** *P* < 0.01, ***P < 0.001, interaction F(1, 32) = 5.493, virus factor F(1, 32) = 2.124, genotype factor F(1, 32) = 15.41, two‐way ANOVA with Tukey post hoc test. N, Hippocampal LTP in WT+vehicle, WT+vehicle+nelfinavir, Ts65Dn+vehicle, Ts65Dn+vehicle+nelfinavir, Ts65Dn+PQBP1, Ts65Dn+PQBP1+nelfinavir mice. Arrows indicate HFS. WT+vehicle *n* = 7; WT+vehicle+nelfinavir *n* = 7; Ts65Dn+vehicle *n* = 8; Ts65Dn+vehicle+nelfinavir *n* = 9; Ts65Dn+PQBP1 *n* = 9; Ts65Dn+PQBP1+nelfinavir *n* = 7. O, Cumulative data of fEPSP slopes at 90 minutes after HFS in WT+vehicle, WT+vehicle+nelfinavir, Ts65Dn+vehicle, Ts65Dn+vehicle+nelfinavir, Ts65Dn+PQBP1, Ts65Dn+PQBP1+nelfinavir mice. WT+vehicle *n* = 7; WT+vehicle+nelfinavir *n* = 7; Ts65Dn+vehicle *n* = 8; Ts65Dn+vehicle+nelfinavir *n* = 9; Ts65Dn+PQBP1 *n* = 9; Ts65Dn+PQBP1+nelfinavir *n* = 7. **** *P* < 0.0001, F(5, 41) = 24.12, one‐way ANOVA with Tukey post hoc test. ANOVA, analysis of variance; DS, Down syndrome; eEF2, eukaryotic elongation factor 2; eEF2K, eukaryotic elongation factor 2 kinase; fEPSP, field excitatory postsynaptic potential; GAPDH, glyceraldehyde 3‐phosphate dehydrogenase; HFS, high‐frequency stimulation; LTP, long‐term potentiation; NOR, novel object recognition; OF, open field; PQBP1, polyglutamine binding protein 1; WT wild type

eEF2K activity can be affected by multiple upstream regulators under various conditions. ^46^, ^47^ Based on previous studies, we systematically examined many potential regulators of eEF2K in whole lysate and synaptosome of hippocampi in WT and Ts65Dn mice, including AMPK, extracellular signal‐regulated kinase (ERK), glycogen synthase kinase 3α/β (GSK3α/β), protein kinase A (PKA), p38 mitogen‐activated protein kinase (p38 MAPK), S6 Kinase 1 (S6K1), and mTORC1. Surprisingly, activities of these molecules were not changed either in whole lysate or synaptosome of hippocampi in Ts65Dn mice compared to WT mice except for GSK3α/β, S6K1, mTORC1, which was further evidenced by its downstream effector eukaryotic translation initiation factor 4E‐bingding protein 1 (4EBP1; Figure S4 and S5 in supporting information). However, increased phosphorylation of GSK3α/β at ser21/9 would indicate reduced GSK3α/β activity and should in turn suppress eEF2K activity and decrease eEF2 phosphorylation, while increased phosphorylation of S6K1 and mTORC1 should also suppress eEF2K activity and decrease eEF2 phosphorylation.^47^, ^48^, ^49^ Thus, none of the activity alterations in these kinases could explain eEF2 hyperphosphorylation in Ts65Dn mice. A more recent study reported that polyglutamine binding protein 1 (PQBP1) could bind eEF2 to protect it from phosphorylation by eEF2K, and inhibition of PQBP1 led to increased eEF2 phosphorylation.^50^ Interestingly, we found that levels of PQBP1 were significantly decreased in the hippocampi of both patients with DS and Ts65Dn mice (Figure 6A and 6B). We next investigated whether upregulation of PQBP1 expression could alleviate DS‐associated eEF2 hyperphosphorylation and cognitive impairment. We developed a recombinant adeno‐associated virus 9 (AAV9) to express the first 173 amino acids of PQBP1 with 3 hemagglutinin (HA) tags and green fluorescent protein (GFP) under the promoter of human synapsin 1 as well as control virus which only expressed GFP, and microinjected the viruses into hippocampi of WT and Ts65Dn mice (Figure 6C and 6D). We started behavioral tests on day 18 and sacrificed the mice on day 35 (Figure 6C). Immunofluorescence imaging showed that viruses were successfully expressed in the bilateral hippocampi (Figure 6D). Western blot of the hippocampal tissues injected with PQBP1 virus also confirmed the expression of PQBP1 with HA tags (Figure S6A in supporting information). Overexpression of PQBP1 in Ts65Dn mice significantly decreased p‐eEF2 level compared to mice injected with vehicle, while overexpression of PQBP1 in WT mice did not change p‐eEF2 level (Figure 6E). Electrophysiology experiments demonstrated that overexpression of PQBP1 in the hippocampus alleviated hippocampal LTP impairment in Ts65Dn mice without affecting LTP performance in WT mice (Figure 6F and 6G). For behavioral tests, PQBP1 overexpression did not affect performance of either WT or Ts65Dn mice in the OF task (Figure 6I and 6J). Remarkably, PQBP1 overexpression improved long‐term recognition memory deficits in Ts65Dn mice (with vehicle injection) assessed by the NOR test (Figure 6K). Additionally, NOR performance of WT mice was not altered with PQBP1 overexpression (Figure 6K). In the training phase of MWM test, performance of Ts65Dn mice injected with PQBP1 virus was not significantly improved compared to the Ts65Dn mice injected with vehicle (repeated ANOVA, *P *> 0.05; Figure 6L). In the probe trial, Ts65Dn mice injected with vehicle virus displayed memory deficits indicated by less target quadrant occupancy compared to WT mice. Importantly, such deficits were rescued with PQBP1 overexpression (Figure 6M). To confirm whether the beneficial effects of PQBP1 overexpression on Ts65Dn mice were mediated by eEF2K signaling, we used an eEF2K agonist, nelfinavir.^51^ First, we confirmed that nelfinavir treatment of the hippocampal slices from WT mice significantly increased p‐eEF2 level, while it did not alter p‐eEF2 level in eEF2K knockout mice, suggesting that nelfinavir could increase eEF2 phosphorylation through activating eEF2K (Figure S6B and S6C). Strikingly, LTP improvement in Ts65Dn mice with PQBP1 overexpression was reversed by nelfinavir treatment (Figure 6N and 6O). Taken together, these results suggested that PQBP1 dysregulation could be involved in DS‐associated synaptic failure and cognitive deficits through its regulation on the eEF2K signaling.

### 3.7 Treatment with small molecule inhibitors of eEF2K rescues cognitive deficits and synaptic plasticity impairment in Ts65Dn mice

**FIGURE 7:**
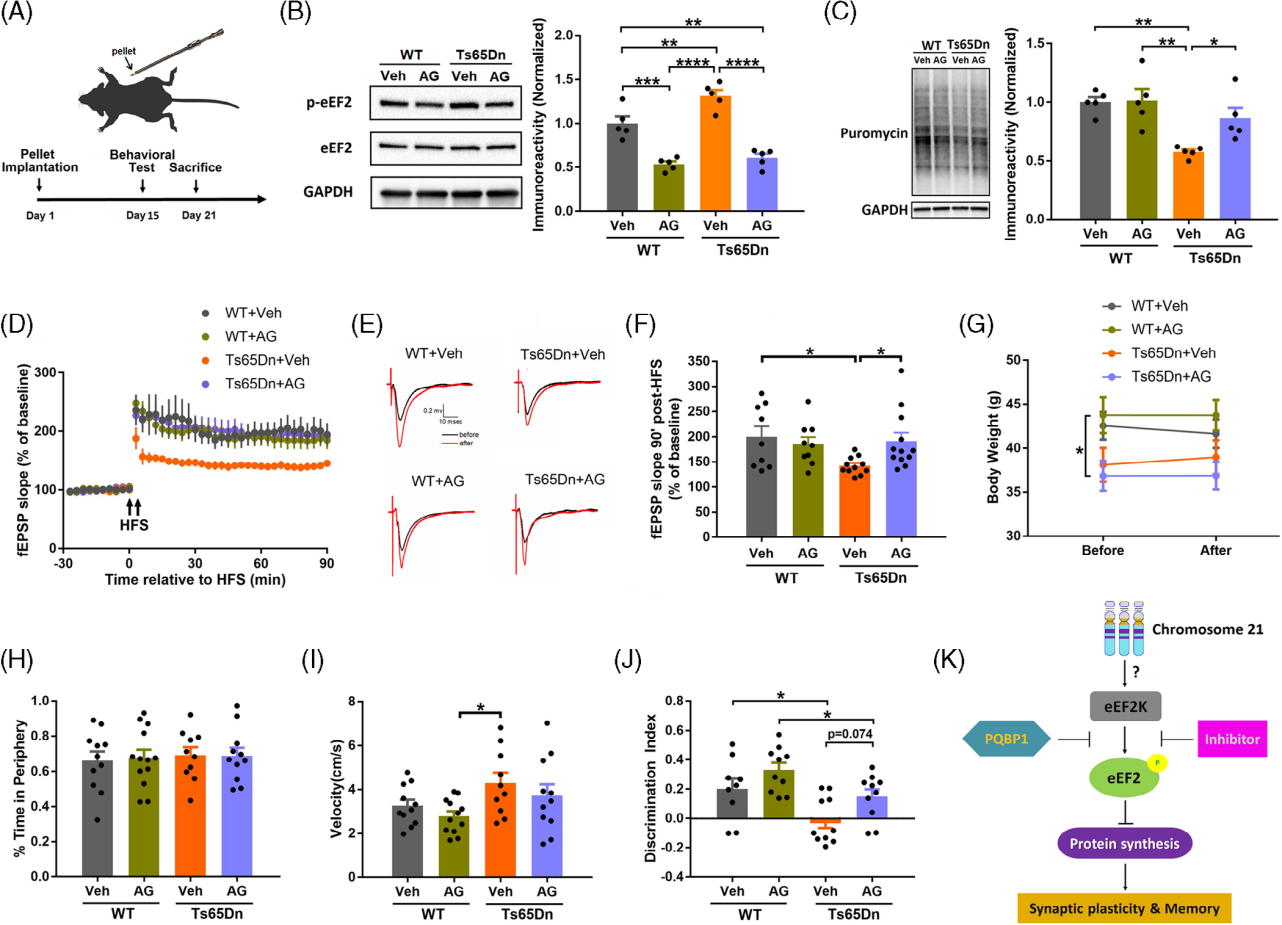
Treatment of eEF2K inhibitors can improve synaptic and cognitive deficits in Ts65Dn mice. A, Schematic diagram of the drug pellet implantation in mice (upper) and timeline of the experiments (bottom). B, Representative western blot images of p‐eEF2, eEF2, and GAPDH in hippocampal tissues from WT+vehicle, WT+AG, Ts65Dn+vehicle, and Ts65Dn+AG mice, and quantification of p‐eEF2 levels in the four groups. *n* = 5 in each group. ** *P* < 0.01, *** *P* < 0.001, **** *P* < 0.0001, interaction F(1, 16) = 4.271, drug factor F(1, 16) = 99.02, genotype factor F(1, 16) = 10.72, two‐way ANOVA with Tukey post hoc test. C, Representative images and quantification of the SUnSET assay in hippocampal tissues from WT+vehicle, WT+AG, Ts65Dn+vehicle, and Ts65Dn+AG mice. *n* = 5 in each group. * *P* < 0.05, ** *P* < 0.01, interaction F(1, 16) = 3.728, drug factor F(1, 16) = 4.529, genotype factor F(1, 16) = 16.24, two‐way ANOVA with Tukey's post hoc test. D, Hippocampal LTP in WT+vehicle, WT+AG, Ts65Dn+vehicle, and Ts65Dn+AG mice. Arrows indicate HFS. WT+vehicle *n* = 9; WT+AG *n* = 9; Ts65Dn+vehicle *n* = 11; Ts65Dn+AG *n* = 12. E, Representative traces before and after HFS in WT+vehicle, WT+AG, Ts65Dn+vehicle and Ts65Dn+AG mice. F, Cumulative data of fEPSP slopes at 90 minutes after HFS in WT+vehicle, WT+AG, Ts65Dn+vehicle, and Ts65Dn+AG mice. WT+vehicle *n* = 9; WT+AG *n* = 9; Ts65Dn+vehicle *n* = 11; Ts65Dn+AG *n* = 12. * *P* < 0.05, interaction F(1, 37) = 4.467, drug factor F(1, 37) = 1.295, genotype factor F(1, 37) = 2.895, two‐way ANOVA with Tukey post hoc test. G, Body weights before and 3 weeks after drug pellet implantation in WT+vehicle, WT+AG, Ts65Dn+vehicle and Ts65Dn+AG mice. WT+vehicle *n* = 8; WT+AG *n* = 8; Ts65Dn+vehicle *n* = 7; Ts65Dn+AG *n* = 7. * *P* < 0.05, interaction F(3, 26) = 1.207, drug factor F(1, 26) < 0.001, genotype factor F(3, 26) = 3.304, two‐way repeated measures ANOVA with Tukey post hoc test. H, Ratio of time spent in the periphery in OF test of WT+vehicle, WT+AG, Ts65Dn+vehicle and Ts65Dn+AG mice. WT+vehicle *n* = 11; WT+AG *n* = 13; Ts65Dn+vehicle *n* = 10; Ts65Dn+AG *n* = 11. Interaction F(1, 41) = 0.0414, *p* = 0.8397; drug factor F(1, 41) = 0.0181, *P* = 0.8936; genotype factor F(1, 41) = 0.1867, *P* = 0.6679; two‐way ANOVA with Tukey post hoc test. I, Velocities of WT+vehicle, WT+AG, Ts65Dn+vehicle, and Ts65Dn+AG mice in OF test. WT+vehicle *n* = 11; WT+AG *n* = 12; Ts65Dn+vehicle *n* = 10; Ts65Dn+AG *n* = 11. * *P* < 0.05, Interaction F(1, 40) = 0.0129, drug factor F(1, 40) = 1.886, genotype factor F(1, 40) = 6.647, two‐way ANOVA with Tukey post hoc test. J, Discrimination index of WT+vehicle, WT+AG, Ts65Dn+vehicle, and Ts65Dn+AG mice in NOR test. WT+vehicle *n* = 9; WT+AG *n* = 10; Ts65Dn+vehicle *n* = 10; Ts65Dn+AG *n* = 11. * *P* < 0.05, ** *P* < 0.01, interaction F(1, 35) = 0.1005, drug factor F(1, 35) = 7.287, genotype factor F(1, 35) = 13.39, two‐way ANOVA with Tukey post hoc test. K, Schematic model based on the main findings of the study. eEF2K signaling was dysregulated in the brain of patients with DS and mouse models, which impaired *de novo* protein synthesis and in turn impaired synaptic plasticity and cognition. PQBP1 was a potential regulator of eEF2K signaling in DS. Suppressing eEF2K activity by genetic or pharmacological approaches could alleviate synaptic dysfunctions and cognitive deficits in DS mice. ANOVA, analysis of variance; DS, Down syndrome; eEF2, eukaryotic elongation factor 2; eEF2K, eukaryotic elongation factor 2 kinase; fEPSP, field excitatory postsynaptic potential; GAPDH, glyceraldehyde 3‐phosphate dehydrogenase; HFS, high‐frequency stimulation; LTP, long‐term potentiation; NOR, novel object recognition; OF, open field; PQBP1, polyglutamine binding protein 1; PSD, postsynaptic density; SUnSET, surface sensing of translation; WT wild type

We went on to investigate the therapeutic potential of eEF2K inhibition for DS‐associated cognitive impairments and synaptic failure by using two structurally distinct small molecules eEF2K inhibitor: A‐484594 (AG) and NH125.^26^, ^47^ First, we conducted ex vivo experiments in Ts65Dn and littermate WT mice with NH125. NH125 treatment of hippocampal slices alleviated LTP impairment in Ts65Dn mice compared to those treated with vehicle, and did not alter LTP in WT mice (Figure S7A‐C in supporting information). Next, we conducted in vivo experiments in Ts65Dn mice as well as in littermate WT mice with eEF2K inhibitor A484954 (AG). AG compound was packed into pellets and implanted subcutaneously to enable the drug to be released gradually. We started behavioral tests on day 15 after pellet implantation and sacrificed the mice afterward (Figure 7A). We first confirmed that treatment of AG decreased p‐eEF2 levels in the hippocampi of both WT and Ts65Dn mice (Figure 7B). Furthermore, defects of *de novo* protein synthesis (assessed by the SUnSET assay) in the hippocampus of Ts65Dn were improved with AG treatment (Figure 7C). Functionally, treatment of AG alleviated hippocampal LTP impairment in Ts65Dn mice without affecting LTP performance in WT mice (Figure 7D‐F). In addition, treatment of AG did not alter the body weights of these mice (Figure 7G). Results from behavioral experiments showed that treatment of AG did not impact the performance in the OF test for either WT or Ts65Dn mice (Figure 7H and 7I). Notably, treatment of AG improved long‐term recognition memory deficits (assessed by NOR test) in Ts65Dn mice (Figure 7J). In brief, treatment of eEF2K inhibitors could decrease eEF2 phosphorylation, boost *de novo* protein synthesis, reverse synaptic impairments, and rescue cognitive deficits in Ts65Dn DS model mice.

## 4 DISCUSSION

Despite wide deployment of prenatal screening and intervention, DS continues to be the leading cause of intellectual disability worldwide, posing significant socioeconomic impacts.^52^, ^53^ With a dramatic increase of life expectancy over the past few decades, aging‐related cognitive impairment becomes a common pathophysiology in people with DS.^54^ Substantial evidence has demonstrated that a homeostasis of protein synthesis is critical for normal cognitive and synaptic function.^10^, ^55^ In this study, we showed that eEF2 phosphorylation was abnormally increased in the brain of patients with DS and mouse models, which was associated with repression of general protein synthesis. Importantly, restoration of eEF2 phosphorylation by suppression of eEF2K could alleviate multiple pathophysiological abnormalities and improve aging‐related cognitive deficits in two different lines of a DS mouse model. Interestingly, our data suggested a role of PQBP1 dysregulation in DS‐associated eEF2 hyperphosphorylation and cognitive deficits. More comprehensive studies in the future are warranted to elucidate the interaction between PQBP1 and eEF2K/eEF2 signaling in the context of DS pathogenesis. Finally, as a proof‐of‐concept study, we showed that the small‐molecule eEF2K inhibitor A484954 could rescue cognitive impairments and synaptic failure in DS model mice, suggesting targeting the eEF2K signaling could be a feasible therapeutic strategy for aging‐related cognitive impairments in DS. Notably, patients with DS older than 40 years usually develop AD‐like brain pathology and dementia syndromes.^56^ Recent studies from us and other groups demonstrated a role of eEF2 hyperphosphorylation in AD pathogenesis.^13^, ^25^, ^26^, ^57^, ^58^

Impaired capacity of *de novo* protein synthesis has been linked to multiple neuronal diseases characterized by cognitive deficits.^10^, ^11^, ^14^, ^59^, ^60^, ^61^ Regulation of protein synthesis has been associated with distinct translational factors involved in each phase of mRNA translation.^62^ Recent studies provided compelling evidence that dysregulation of the integrated stress response (ISR) signaling was involved in DS pathophysiology.^15^, ^63^ Interestingly, it was demonstrated in the Ts65Dn mouse model that DS‐associated synaptic and cognitive impairments can be improved by boosting general mRNA translation through regulation of the eukaryotic translation initiation factor eIF2, a critical player mediating the ISR.^15^ Despite decades of intensive research in the neuroscience field, identities of “memory proteins” of “plasticity‐related proteins” (PRPs) remain elusive.^64^ Besides protein synthesis, dysregulation of protein degradation has been implicated in DS based on proteomic analysis in human tissue.^65^ Taken together, we would propose that the restoration of overall protein synthesis capacity and homeostasis could be an alternative and feasible strategy to alleviate DS‐associated synaptic failure and cognitive deficits.

From a translational perspective, inhibition of eEF2K and eEF2 phosphorylation can be an appealing therapeutic approach for aging‐related cognitive deficits in DS. Unlike most of the protein kinases including serine/threonine kinases and tyrosine kinases, eEF2K is one of the few “alpha‐kinases” whose catalytic domains are distinct from the conventional kinases.^66^ Further, there is a 1:1 ratio for eEF2K and eEF2 phosphorylation (Thr^56^), that is, eEF2 is the only known substrate for eEF2K and eEF2K is the only known kinase for eEF2. All these features can help improve the selectivity/specificity and reduce the off‐target effects of the small‐molecule eEF2K inhibitors with proper design. Moreover, inhibition of eEF2K could be a “safe” therapeutic strategy. Transgenic mice with global homozygous knockout of eEF2K are physiologically normal during development.^67^ And treatment of the eEF2K inhibitor does not induce any adverse effects in WT mice.^26^ Patients with neurological disorders usually need to take medicine over a long period of time, and the safety issue is often a prime factor to be considered for choosing medicine, particularly for the elderly.

Previous studies mainly from non‐neuronal systems indicated that eEF2K could be regulated by multiple upstream signaling pathways.^47^ In this study, we explored the possible upstream regulators of eEF2K signaling in DS. To our surprise, the data suggested that eEF2 hyperphosphorylation in the DS brain is unlikely to be associated with any of these canonical upstream regulators. We checked a recently identified regulator of eEF2K signaling, PQBP1, and found that PQBP1 was indeed dysregulated both in the hippocampi of patients with DS and mouse models. Importantly, restoration of PQBP1 expression in the hippocampi of DS mice could alleviate synaptic dysfunction and cognitive deficits. Interestingly, dysregulation of PQBP1 was also found in AD and was under the control of serine/arginine repetitive matrix 2 (SRRM2). Restoration of PQBP1 expression could rescue AD‐related pathologies and cognitive deficits in mouse models.^68^ PQBP1 was a causative gene for intellectual disability, which affected splicing patterns of many synapse‐related genes.^69^ It is appealing for future studies to elucidate the roles of PQBP1 in DS including whether and how dysregulation of PQBP1 affects signaling pathways other than eEF2K.

What are the downstream effectors (other than general protein synthesis) that are associated with the beneficial phenotypes observed in DS mice with eEF2K inhibition and eEF2 de‐phosphorylation? Previous work from our lab showed that either knockdown or knockout of eEF2K in AD mouse models could alleviate cognitive deficits in a protein synthesis‐dependent manner and possibly through the NRF2‐mediated antioxidant response.^25^, ^67^ In another study, eEF2K knockout in the dentate gyrus excitatory neurons could enhance neurogenesis and upregulate neurogenesis‐related proteins.^70^ Consistent with these studies, a study found that suppression of eEF2K in neurons could upregulate the synthesis of microtubule‐related proteins, which are critical components of synaptic structure.^71^ With bioinformatic analysis on proteomics data, we found that suppression of eEF2K signaling in DS mice could promote protein synthesis‐related pathways and synaptogenesis‐related proteins such as ADGRB3, which also is known to be involved in synaptogenesis.^45^ Future comprehensive studies are required to elucidate whether eEF2K suppression can improve neurogenesis and/or synaptogenesis in DS, and potentially other AD‐related dementia syndromes.

## CONFLICT OF INTEREST STATEMENT

The authors have no conflicts of interest to report. Author disclosures are available in the supporting information.

## CONSENT STATEMENT

No human subjects were included in the study. Consent was not necessary for the *post mortem* human sample study.

## Supporting information

Supporting information

Supporting information

## References

[1] Megarbane A , Noguier F , Stora S , et al. The intellectual disability of trisomy 21: differences in gene expression in a case series of patients with lower and higher IQ. Eur J Hum Genet. 2013;21:1253‐1259. 23422941 10.1038/ejhg.2013.24 PMC3798834

[2] Rafii MS , Kleschevnikov AM , Sawa M , Mobley WC . Down syndrome. Handb Clin Neurol. 2019;167:321‐336. 31753140 10.1016/B978-0-12-804766-8.00017-0

[3] Lott IT , Dierssen M . Cognitive deficits and associated neurological complications in individuals with Down's syndrome. Lancet Neurol. 2010;9:623‐633. 20494326 10.1016/S1474-4422(10)70112-5

[4] Fortea J , Zaman SH , Hartley S , Rafii MS , Head E , Carmona‐Iragui M . Alzheimer's disease associated with Down syndrome: a genetic form of dementia. Lancet Neurol. 2021;20:930‐942. 34687637 10.1016/S1474-4422(21)00245-3 PMC9387748

[5] Lott IT , Head E . Dementia in Down syndrome: unique insights for Alzheimer disease research. Nat Rev Neurol. 2019;15:135‐147. 30733618 10.1038/s41582-018-0132-6 PMC8061428

[6] Head E , Lott IT , Wilcock DM , Lemere CA . Aging in Down syndrome and the development of Alzheimer's disease neuropathology. Curr Alzheimer Res. 2016;13:18‐29. 26651341 10.2174/1567205012666151020114607 PMC4948181

[7] Snyder HM , Bain LJ , Brickman AM , et al. Further understanding the connection between Alzheimer's disease and Down syndrome. Alzheimers Dement. 2020;16:1065‐1077. 32544310 10.1002/alz.12112 PMC8865308

[8] Sutton MA , Schuman EM . Dendritic protein synthesis, synaptic plasticity, and memory. Cell. 2006;127:49‐58. 17018276 10.1016/j.cell.2006.09.014

[9] Klann E , Dever TE . Biochemical mechanisms for translational regulation in synaptic plasticity. Nat Rev Neurosci. 2004;5:931‐942. 15550948 10.1038/nrn1557

[10] Sossin WS , Costa‐Mattioli M . Translational control in the brain in health and disease. Cold Spring Harb Perspect Biol. 2019:11. 10.1101/cshperspect.a032912 PMC6671938 30082469

[11] Lepeta K , Lourenco MV , Schweitzer BC , et al. Synaptopathies: synaptic dysfunction in neurological disorders—A review from students to students. J Neurochem. 2016;138:785‐805. 27333343 10.1111/jnc.13713 PMC5095804

[12] Kapur M , Monaghan CE , Ackerman SL . Regulation of mRNA translation in neurons‐a matter of life and death. Neuron. 2017;96:616‐637. 29096076 10.1016/j.neuron.2017.09.057 PMC5693308

[13] Ma T . Roles of eukaryotic elongation factor 2 kinase (eEF2K) in neuronal plasticity, cognition, and Alzheimer disease. J Neurochem. 2021. [Epub ahead of print]. 10.1111/jnc.15541 PMC9117558 34796967

[14] Ma T . Dysregulation of neuronal protein synthesis in Alzheimer's diseas. The Oxford Handbook of Neuronal Protein Synthesis Edited by Wayne Sossin. 2020:533‐550. Oxford University Press.

[15] Zhu PJ , Khatiwada S , Cui Y , et al. Activation of the ISR mediates the behavioral and neurophysiological abnormalities in Down syndrome. Science. 2019;366:843‐849. 31727829 10.1126/science.aaw5185 PMC7299149

[16] Browne GJ , Proud CG . Regulation of peptide‐chain elongation in mammalian cells. Eur J Biochem. 2002;269:5360‐5368. 12423334 10.1046/j.1432-1033.2002.03290.x

[17] Kenney JW , Moore CE , Wang X , Proud CG . Eukaryotic elongation factor 2 kinase, an unusual enzyme with multiple roles. Adv Biol Regul. 2014;55:15‐27. 24853390 10.1016/j.jbior.2014.04.003

[18] Biever A , Donlin‐Asp PG , Schuman EM . Local translation in neuronal processes. Curr Opin Neurobiol. 2019;57:141‐148. 30861464 10.1016/j.conb.2019.02.008

[19] Proud CG . Regulation and roles of elongation factor 2 kinase. Biochem Soc Trans. 2015;43:328‐332. 26009171 10.1042/BST20140323

[20] Ryazanov AG , Davydova EK . Mechanism of elongation factor 2 (EF‐2) inactivation upon phosphorylation. Phosphorylated EF‐2 is unable to catalyze translocation. FEBS Lett. 1989;251:187‐190. 2753158 10.1016/0014-5793(89)81452-8

[21] Ryazanov AG , Pavur KS , Dorovkov MV . Alpha‐kinases: a new class of protein kinases with a novel catalytic domain. Curr Biol. 1999;9:R43‐45. 10.1016/s0960-9822(99)80006-2 10021370

[22] Horman S , Browne GJ , Krause U , et al. Activation of AMP‐activated protein kinase leads to the phosphorylation of elongation factor 2 and an inhibition of protein synthesis. Curr Biol. 2002;12:1419‐1423. 12194824 10.1016/s0960-9822(02)01077-1

[23] Hawley SA , Ross FA , Gowans GJ , Tibarewal P , Leslie NR , Hardie DG . Phosphorylation by Akt within the ST loop of AMPK‐α1 down‐regulates its activation in tumour cells. Biochem J. 2014;459:275‐287. 24467442 10.1042/BJ20131344 PMC4052680

[24] Hardie DG . The AMP‐activated protein kinase pathway–new players upstream and downstream. J Cell Sci. 2004;117:5479‐5487. 15509864 10.1242/jcs.01540

[25] Beckelman BC , Yang W , Kasica NP , et al. Genetic reduction of eEF2 kinase alleviates pathophysiology in Alzheimer's disease model mice. J Clin Invest. 2019;129:820‐833. 30667373 10.1172/JCI122954 PMC6355242

[26] Kasica NP , Zhou X , Yang Q , et al. Antagonists targeting eEF2 kinase rescue multiple aspects of pathophysiology in Alzheimer's disease model mice. J Neurochem. 2022;160:524‐539. 34932218 10.1111/jnc.15562 PMC8902702

[27] Kameshima S , Kazama K , Okada M , Yamawaki H . Eukaryotic elongation factor 2 kinase mediates monocrotaline‐induced pulmonary arterial hypertension via reactive oxygen species‐dependent vascular remodeling. Am J Physiol Heart Circ Physiol. 2015;308:H1298‐1305. 25770246 10.1152/ajpheart.00864.2014

[28] Reeves RH , Irving NG , Moran TH , et al. A mouse model for Down syndrome exhibits learning and behaviour deficits. Nat Genet. 1995;11:177‐184. 7550346 10.1038/ng1095-177

[29] Schmidt EK , Clavarino G , Ceppi M , Pierre P . SUnSET, a nonradioactive method to monitor protein synthesis. Nat Methods. 2009;6:275‐277. 19305406 10.1038/nmeth.1314

[30] Li Z , Yu T , Morishima M , et al. Duplication of the entire 22.9 Mb human chromosome 21 syntenic region on mouse chromosome 16 causes cardiovascular and gastrointestinal abnormalities. Hum Mol Genet. 2007;16:1359‐1366. 17412756 10.1093/hmg/ddm086

[31] Schafe GE , Nadel NV , Sullivan GM , Harris A , LeDoux JE . Memory consolidation for contextual and auditory fear conditioning is dependent on protein synthesis, PKA, and MAP kinase. Learn Mem. 1999;6:97‐110. 10327235 PMC311283

[32] Tsokas P , Grace EA , Chan P , et al. Local protein synthesis mediates a rapid increase in dendritic elongation factor 1A after induction of late long‐term potentiation. J Neurosci. 2005;25:5833‐5843. 15958750 10.1523/JNEUROSCI.0599-05.2005 PMC6724870

[33] Seibenhener ML , Wooten MC . Use of the open field maze to measure locomotor and anxiety‐like behavior in mice. J Vis Exp. 2015:e52434. 25742564 10.3791/52434 PMC4354627

[34] Leger M , Quiedeville A , Bouet V , et al. Object recognition test in mice. Nat Protoc. 2013;8:2531‐2537. 24263092 10.1038/nprot.2013.155

[35] Hering H , Sheng M . Dendritic spines: structure, dynamics and regulation. Nat Rev Neurosci. 2001;2:880‐888. 11733795 10.1038/35104061

[36] Rochefort NL , Konnerth A . Dendritic spines: from structure to in vivo function. EMBO Rep. 2012;13:699‐708. 22791026 10.1038/embor.2012.102 PMC3410382

[37] Day SM , Yang W , Wang X , et al. Glucagon‐like peptide‐1 cleavage product improves cognitive function in a mouse model of Down syndrome. eNeuro. 2019;6. 10.1523/ENEURO.0031-19.2019 PMC6520642 31040160

[38] Yang W , Zhou X , Zimmermann HR , Ma T . Brain‐specific suppression of AMPKα2 isoform impairs cognition and hippocampal LTP by PERK‐mediated eIF2α phosphorylation. Mol Psychiatry. 2021;26:1880‐1897. 32366952 10.1038/s41380-020-0739-z PMC8054310

[39] Risher WC , Ustunkaya T , Singh Alvarado J , Eroglu C . Rapid Golgi analysis method for efficient and unbiased classification of dendritic spines. PLoS One. 2014;9:e107591. 25208214 10.1371/journal.pone.0107591 PMC4160288

[40] Yang W , Zhou X , Zimmermann HR , Ma T . Brain‐specific suppression of AMPKalpha2 isoform impairs cognition and hippocampal LTP by PERK‐mediated eIF2alpha phosphorylation. Mol Psychiatry. 2021;26:1880‐1897. 32366952 10.1038/s41380-020-0739-z PMC8054310

[41] Uguagliati B , Al‐Absi AR , Stagni F , et al. Early appearance of developmental alterations in the dendritic tree of the hippocampal granule cells in the Ts65Dn model of Down syndrome. Hippocampus. 2021;31:435‐447. 33464704 10.1002/hipo.23303

[42] Nicoll RA . A brief history of long‐term potentiation. Neuron. 2017;93:281‐290. 28103477 10.1016/j.neuron.2016.12.015

[43] Siarey RJ , Stoll J , Rapoport SI , Galdzicki Z . Altered long‐term potentiation in the young and old Ts65Dn mouse, a model for Down Syndrome. Neuropharmacology. 1997;36:1549‐1554. 9517425 10.1016/s0028-3908(97)00157-3

[44] Zecha J , Satpathy S , Kanashova T , et al. TMT labeling for the masses: a robust and cost‐efficient, in‐solution labeling approach. Mol Cell Proteomics. 2019;18:1468‐1478. 30967486 10.1074/mcp.TIR119.001385 PMC6601210

[45] Sigoillot SM , Iyer K , Binda F , et al. The secreted protein C1QL1 and its receptor BAI3 control the synaptic connectivity of excitatory inputs converging on. Cerebellar Purkinje Cells Cell Rep. 2015;10:820‐832. 25660030 10.1016/j.celrep.2015.01.034

[46] Taha E , Gildish I , Gal‐Ben‐Ari S , Rosenblum K . The role of eEF2 pathway in learning and synaptic plasticity. Neurobiol Learn Mem. 2013;105:100‐106. 23742918 10.1016/j.nlm.2013.04.015

[47] Liu R , Proud CG . Eukaryotic elongation factor 2 kinase as a drug target in cancer, and in cardiovascular and neurodegenerative diseases. Acta Pharmacol Sin. 2016;37:285‐294. 26806303 10.1038/aps.2015.123 PMC4775846

[48] Wang X , Regufe da Mota S , Liu R , et al. Eukaryotic elongation factor 2 kinase activity is controlled by multiple inputs from oncogenic signaling. Mol Cell Biol. 2014;34:4088‐4103. 25182533 10.1128/MCB.01035-14 PMC4248706

[49] Wang X , Li W , Williams M , Terada N , Alessi DR , Proud CG . Regulation of elongation factor 2 kinase by p90(RSK1) and p70 S6 kinase. Embo j. 2001;20:4370‐4379. 11500364 10.1093/emboj/20.16.4370 PMC125559

[50] Shen Y , Zhang ZC , Cheng S , et al. PQBP1 promotes translational elongation and regulates hippocampal mGluR‐LTD by suppressing eEF2 phosphorylation. Mol Cell. 2021;81:1425‐1438. e10. 33662272 10.1016/j.molcel.2021.01.032

[51] Smith PR , Loerch S , Kunder N , Stanowick AD , Lou TF , Campbell ZT . Functionally distinct roles for eEF2K in the control of ribosome availability and p‐body abundance. Nat Commun. 2021;12:6789. 34815424 10.1038/s41467-021-27160-4 PMC8611098

[52] Loane M , Morris JK , Addor MC , et al. Twenty‐year trends in the prevalence of Down syndrome and other trisomies in Europe: impact of maternal age and prenatal screening. Eur J Hum Genet. 2013;21:27‐33. 22713804 10.1038/ejhg.2012.94 PMC3522199

[53] Chen L , Wang L , Wang Y , et al. Global, regional, and national burden and trends of Down syndrome from 1990 to 2019. Front Genet. 2022;13:908482. 35910218 10.3389/fgene.2022.908482 PMC9337874

[54] Fortea J , Vilaplana E , Carmona‐Iragui M , et al. Clinical and biomarker changes of Alzheimer's disease in adults with Down syndrome: a cross‐sectional study. Lancet. 2020;395:1988‐1997. 32593336 10.1016/S0140-6736(20)30689-9 PMC7322523

[55] Giandomenico SL , Alvarez‐Castelao B , Schuman EM . Proteostatic regulation in neuronal compartments. Trends Neurosci. 2022;45:41‐52. 34489114 10.1016/j.tins.2021.08.002

[56] Ballard C , Mobley W , Hardy J , Williams G , Corbett A . Dementia in Down's syndrome. Lancet Neurol. 2016;15:622‐636. 27302127 10.1016/S1474-4422(16)00063-6

[57] Kasica NP , Zhou X , Jester HM , et al. Homozygous knockout of eEF2K alleviates cognitive deficits in APP/PS1 Alzheimer's disease model mice independent of brain amyloid β pathology. Front Aging Neurosci. 2022;14:959326. 36158543 10.3389/fnagi.2022.959326 PMC9500344

[58] Jan A , Jansonius B , Delaidelli A , et al. eEF2K inhibition blocks Aβ42 neurotoxicity by promoting an NRF2 antioxidant response. Acta Neuropathol. 2017;133:101‐119. 27752775 10.1007/s00401-016-1634-1

[59] Abisambra JF , Jinwal UK , Blair LJ , et al. Tau accumulation activates the unfolded protein response by impairing endoplasmic reticulum‐associated degradation. J Neurosci. 2013;33:9498‐9507. 23719816 10.1523/JNEUROSCI.5397-12.2013 PMC3733249

[60] Lourenco MV , Clarke JR , Frozza RL , et al. TNF‐α mediates PKR‐dependent memory impairment and brain IRS‐1 inhibition induced by Alzheimer's β‐amyloid oligomers in mice and monkeys. Cell Metab. 2013;18:831‐843. 24315369 10.1016/j.cmet.2013.11.002

[61] Beckelman BC , Day S , Zhou X , et al. Dysregulation of elongation factor 1A expression is correlated with synaptic plasticity impairments in Alzheimer's disease. J Alzheimers Dis. 2016;54:669‐678. 27567813 10.3233/JAD-160036 PMC5439429

[62] Hershey JW , Sonenberg N , Mathews MB . Principles of translational control: an overview. Cold Spring Harb Perspect Biol. 2012;4. 10.1101/cshperspect.a011528 PMC3504442 23209153

[63] Lanzillotta C , Zuliani I , Tramutola A , et al. Chronic PERK induction promotes Alzheimer‐like neuropathology in Down syndrome: insights for therapeutic intervention. Prog Neurobiol. 2021;196:101892. 32795489 10.1016/j.pneurobio.2020.101892

[64] Okuda K , Højgaard K , Privitera L , Bayraktar G , Takeuchi T . Initial memory consolidation and the synaptic tagging and capture hypothesis. Eur J Neurosci. 2020. 10.1111/ejn.14902 32649022

[65] Liu Y , Borel C , Li L , et al. Systematic proteome and proteostasis profiling in human Trisomy 21 fibroblast cells. Nat Commun. 2017;8:1212. 29089484 10.1038/s41467-017-01422-6 PMC5663699

[66] Drennan D , Ryazanov AG . Alpha‐kinases: analysis of the family and comparison with conventional protein kinases. Prog Biophys Mol Biol. 2004;85:1‐32. 15050379 10.1016/S0079-6107(03)00060-9

[67] Kasica NP , Zhou X , Jester HM , et al. Homozygous knockout of eEF2K alleviates cognitive deficits in APP/PS1 Alzheimer's disease model mice independent of brain amyloid beta pathology. Front Aging Neurosci. 2022;14:959326. 36158543 10.3389/fnagi.2022.959326 PMC9500344

[68] Tanaka H , Kondo K , Chen X , et al. The intellectual disability gene PQBP1 rescues Alzheimer's disease pathology. Mol Psychiatry. 2018;23:2090‐2110. 30283027 10.1038/s41380-018-0253-8 PMC6250680

[69] Tanaka H , Okazawa H . PQBP1: the key to intellectual disability, neurodegenerative diseases, and innate immunity. Int J Mol Sci. 2022:23. 35682906 10.3390/ijms23116227 PMC9180999

[70] Taha E , Patil S , Barrera I , et al. eEF2/eEF2K pathway in the mature dentate gyrus determines neurogenesis level and cognition. Curr Biol. 2020;30:3507‐3521. e7. 32707059 10.1016/j.cub.2020.06.061

[71] Kenney JW , Genheden M , Moon KM , Wang X , Foster LJ , Proud CG . Eukaryotic elongation factor 2 kinase regulates the synthesis of microtubule‐related proteins in neurons. J Neurochem. 2016;136:276‐284. 26485687 10.1111/jnc.13407 PMC4843953

