# Supplementary material for: Suppression of eEF2 phosphorylation alleviates synaptic failure and cognitive deficits in mouse models of Down syndrome
Source: Alzheimers Dement. 2024 Jun 27;20(8):5357–74. doi: 10.1002/alz.13916 (PMC11350057; doi:10.1002/alz.13916)
Supplement: Supplementary file 2 — Supporting information [file ALZ-20-5357-s001.docx]

**Supplementary Files**

**Supplementary Materials**

**Materials and Methods**

**Synaptosome preparation**

Dounce tissue grinder and synaptic protein extraction reagent (Thermo scientific, catalog # 87793) with protease and phosphatase inhibitors were pre-cold on ice. Mice were sacrificed by cervical dislocation. Bilateral hippocampi were dissected and placed in the grinder with 250μl extraction reagent. The tissue was homogenized on ice with 10 slow strokes and transferred to a centrifuge tube. The tube was centrifuged at 1200g for 10 min at 4^o^C and the supernant was transferred to a new tube. The supernant was further centrifuged at 15000g for 20min at 4^o^C. The pellet of this centrifuge was synaptosome and the supernant was the whole lysate. The pellet was suspended in lysis buffer with protease and phosphatase inhibitors for further experiments.

**Hippocampal slice preparation**

Mice were sacrificed by cervical dislocation and whole brains were taken out. Acute 400μm transverse hippocampal slices were prepared using a Leica VT1200S vibratome as described before(*67*). Slices were then maintained at room temperature before experimentation for 2 hours in artificial cerebrospinal fluid (ACSF) containing the following: 118mM NaCl, 3.5mM KCl, 2.5mM CaCl_2_, 1.3mM MgSO_4_, 1.25mM NaH_2_PO_4_, 5mM NaHCO_3_, and 15mM glucose, bubbled with 95% O_2_/5% CO_2_.

**SUnSET assay**

Acute 400 µm transverse hippocampal slices were prepared and maintained at room temperature for at least 2 hours in ACSF bubbled with 95% O_2_/5% CO_2_. Then the slices were transferred to bubbled ACSF with puromycin (0.5 µg/ml) for 1 hour at 32°C. Slices were then flash-frozen on dry ice for Western blot. Puromycin-labeled proteins were detected using the mouse monoclonal antibody 12D10 (1:1000, EMD Millipore, catalog # MABE343). Protein synthesis levels were determined by total lane density from 10 kDa to 250 kDa.

**Open field (OF) and Novel object recognition (NOR) test**

Mice were handled for at least 5 days prior to behavioral tests and habituated to the testing facility for an hour prior to experimentation. In OF test, mice were placed in an opaque plastic chamber and allowed to explore for 15 min. The light intensity is around 600 lux. Time spent in the center and periphery of the chamber was measured and calculated as a percentage of total time. Distance moved and velocity were measured using EthoVision XT Tracking Software (Noldus Information Technology). Following OF, mice were placed in the same chamber to get familiarized with two identical objects and allowed to explore for 5 min. Twenty-four hours after familiarization, mice were tested in the same chamber for 5 min with one object replaced with a novel object. All objects were randomly assigned to mice, and the placement of novel objects was counter balanced. Time spent with each object was measured and Discrimination index was calculated as follows: (interaction time with novel object – interaction time with familiar object) / total interaction time. Discrimination index around 0 indicates memory impairment. Time with objects was measured both manually and using EthoVision XT Tracking Software. Mice with a total interaction time of less than 10s were excluded from analysis. Data collection and analysis were performed blinded.

**Morris water maze (MWM) and visible platform (VP) test**

MWM was performed as previously described(*68*). Briefly, the mice were trained to find a hidden platform in a water maze and the paradigm consisted of 4 trials (60s maximum, 15min interval) per day for 5 consecutive days. Escape latency was recorded for each trial and a mean was calculated based on the 4 trials for each training day. A probe trial was performed 2 hours following training on the fifth day. Trajectories, time spent in each maze quadrant, velocity, and distance moved were recorded using EthoVision XT software (Noldus Information Technology) during the probe trial. The visible platform test consisted of 4 trials (60s maximum, 15min interval) per day for 2 consecutive days, with the escape platform marked by a visible cue and moved randomly among 4 locations. Escape latency was recorded for each trial and a mean was calculated based on the 4 trials for each day. Data collection and analysis were performed blinded.

**Virus vectors**

Virus vectors were provided by Vector Biolabs (Malvern, PA). Briefly, a plasmid containing the cDNA of the first 173 amino acids of PQBP1 with 3 HA tags in the N-terminus under the promoter of hSYN and GFP with an upstream internal ribosome entry site (IRES) was packaged into AAV9 vector. Control vector contained only GFP cDNA under the same promoter. The titer of the virus vectors were about 1 x 10^13^ gc/ml. Stocks of the virus vectors were aliquoted upon reception and stored in -80^o^C.

**Stereotactic injection**

Mouse was anesthetized using isoflurane and shaved off on the head. Ophthalmic gel was applied to eyes to prevent cornea injury. Analgesic ketoprofen (2-3 mg/kg/d) was injected subcutaneously around surgery site. Once adequately sedated, the mouse was fixed on a stereotactic platform with continuous isoflurane inhalation. Meanwhile, the mouse was placed on a heating pat and monitored for its temperature, breath and response. After sanitization with iodophor, scalp of the mouse was cut to expose skull. Coordinates of the drilling sites was predetermined according to the injecting site (AP -2.40, ML ±2.30, DV -2.00, relative to bregma). After drilling the holes in skull, 1μl of virus vector was microinjected into each hippocampus at a speed of 0.1 μl/min and the needle was kept there for another 10min. 2% Lidocaine was applied to the exposed skull before the scalp was sewed by surgical adhesive. The mouse was then returned to the cage and injected with ketoprofen (2-3 mg/kg/d) around surgery site for 3 days and monitored for another 10 days to check if there was any infection or signs of pain and distress after surgery.

**Immuno-labeling electron microscopy**

Mice were anesthetized using 2,2,2-Tribromoethanol and transcardially perfused with cold (4°C) 4% PFA, 0.2% glutaraldehyde, 2 mM CaCl2, and 4 mM MgSO4 in 0.1 M cacodylate buffer (pH 7.4). At the Electron Microscopy Core at Augusta University, fixed mouse brains were cut into 125µm thick sections using a Leica VT1000 vibratome. Slices containing brain region of interest were chosen and incubated with 0.1% NaBH_4_ in PBS for 30min and then rinsed in PBS for 3 × 10min. Slices were permeabilized with 0.05% Triton-X-100 in PBS for 30min and blocked with Aurion blocking solution (Electron Microscopy Sciences, PA) for 30min. After washing with Aurion BSA-C solution (Electron Microscopy Sciences, PA) for 2 x 10min, slices were incubated with p-eEF2 antibody (1:2000, Cell Signaling, catalog # 2331) in BSA-C solution at 4°C overnight on a shaker. Slices were then washed in BSA-C solution for 6 x 10min, and incubated with Aurion Ultrasmall Gold reagent (1:200, Electron Microscopy Sciences, PA) in BSA-C solution at 4°C overnight on a shaker. After washed in BSA-C solution for 6 x 10min and PBS for 2 x 10min, slices were postfixed in 2% glutaraldehyde in PBS for 15min and finally washed with distilled water for 4 x 10min. To obtain desired gold particle growth, slices was incubated with GoldEnhance EM (Nanoprobes, NY) according to manufacturer's directions for 2 hours on a shaker. After enhancement, slices were washed thoroughly in cold distilled water for at least 6 x 10min to stop gold enhancement. Slices were then placed in 2% OsO_4_ in 0.1M cacodylate buffer (pH 7.4) for 1 hr. After washed 4 × 10min in cacodylate buffer and 4 × 10 min in distilled water, slices were dehydrated in a graded ethanol series (25%, 50%, 70%, 80%, 95% x 2 times and 100% x 3 times) for 15min each. Slices were then placed in a 1:1 mixture of 100% ethanol and propylene oxide for 30min and followed by 3 changes of propylene oxide for 15min each. After that, slices were incubated in a 1:1 mixture of propylene oxide and Embed 812/Araldite Epoxy Resin (Electron Microscopy Sciences, PA) overnight, then incubated in 3 changes of Embed 812/Araldite Epoxy Resin the next day. Finally, slices were polymerized at 60°C overnight in an embedding oven. Ultrathin sections (65nm) were cut on a Leica UC7 ultramicrotome and mounted on Synaptek slot grids coated with 1.2% Pioloform (Electron Microscopy Sciences, PA). Sections were imaged on a JEOL 1400Flash transmission electron microscope (JEOL, MA) at 120 kV. Images were acquired with a Gatan OneView digital camera (Gatan Inc., CA).

**Western blot**

Tissues were sonicated in lysis buffer with protease and phosphatase inhibitors. Total protein concentrations of samples were determined by BCA assay. Samples containing equal amount of proteins were loaded on 4%–15% Tris-glycine SDS-PAGE gels (Bio-Rad) for standard gel electrophoresis. Following transfer, nitrocellulose membranes were blocked for 15min in SuperBlock TBS Blocking Buffer (Thermo Scientific). All primary and secondary antibodies were diluted in 5% milk/TBST or 5% BSA/TBST. Blots were probed with primary antibodies for p-eEF2 (1:1000, Cell Signaling, catalog # 2331), eEF2 (1:1000, Cell Signaling, catalog # 2332), GAPDH (1:5000, Cell Signaling, catalog # 2118), PQBP1 (1:1000, Abcam, catalog # 100797), p-AMPKα(Thr172) (1:1000, Cell Signaling, catalog # 2535), AMPKα (1:1000, Cell Signaling, catalog # 5832), p-ERK (1:1000, Cell Signaling, catalog # 9101), ERK (1:1000, Cell Signaling, catalog # 9102), p-GSK3α/β (1:1000, Cell Signaling, catalog # 9331), GSK3α/β (1:1000, Cell Signaling, catalog # 5676), p-PKA (1:1000, Cell Signaling, catalog # 5661), PKA (1:1000, RandD, catalog # MAB4175), p-p38 MAPK (1:1000, Cell Signaling, catalog # 4511), p38 MAPK (1:1000, Cell Signaling, catalog # 8690), p-mTORC1(Ser2448) (1:1000, Cell Signaling, catalog # 5536), p-mTORC1(Ser2481) (1:1000, Cell Signaling, catalog # 2974), mTORC1 (1:1000, Cell Signaling, catalog # 2983), p-S6K1 (1:1000, Cell Signaling, catalog # 9234), S6K1 (1:1000, Cell Signaling, catalog # 2708), p-4eBP1 (1:1000, Cell Signaling, catalog # 2855), 4eBP1 (1:1000, Cell Signaling, catalog # 9644), HA tag (1:1000, Abcam, catalog # 9110), ADGRB3 (1:1000, Invitrogen, catalog # PA5-67719). Following primary antibody incubation, blots were then exposed to secondary antibodies, either goat anti-rabbit IgG (H+L)-HRP conjugate (1:5000, Bio-Rad, catalog 170-6515) or goat anti-mouse IgG (H+L)-HRP conjugate (1:5000, Bio-Rad, catalog 170-6516). Proteins were visualized using the ChemiDoc Imaging System (Bio-Rad). Densitometric analysis was performed using Bio-Rad ImageLab and ImageJ software. Phospho-proteins were normalized to total protein, and total proteins were normalized to housekeeping protein GAPDH.

**Postmortem human tissue immunohistochemistry**

Postmortem brain tissue sections from patients were prepared at the University of Washington. Brains were fixed in 10% neutral buffered formalin. Brain tissues were embedded in paraffin and sectioned at 5 µm thickness and mounted on positively charged slides. Sections were baked for 30min at 60°C before staining, and then deparaffinized in xylene and rehydrated through a graded alcohol series. Slides were boiled in citrate buffer (pH 6.0) for 10min to retrieve antigen. Endogenous peroxidase activity was blocked using 3% hydrogen peroxide for 15min. Slides were then incubated in a humid chamber in primary antibody for p-eEF2 (1:500, Cell Signaling, catalog # 2331) overnight at 4°C. Sections were then incubated in biotinylated rabbit secondary antibody (1: 200; Vector Labs, PI-1000) for 30min at room temperature followed by Vectastain Elite ABC Reagent (Vector Labs, PK-4000) for another 30min. Primary and secondary antibodies and ABC reagent were diluted in 1% goat serum in PBS. Diaminobenzidine (DAB) was diluted in the diluent provided by the kit as working solution. Sections were developed in DAB working solution for 15min at room temperature. Slides were counterstained using Mayer’s hematoxylin. Negative controls were incubated in 1% goat serum in PBS as the primary antibody. Sections were dehydrated in an alcohol series and cleared with xylene, coverslipped, and dried overnight. Slides were imaged on a Keyence BZ-X710 microscope.

**Immunofluorescence and confocal microscopy**

Mouse hippocampal slices were fixed overnight in ice-cold 4% paraformaldehyde in PBS. Fixed free-floating sections were further subsectioned to 60 μm using a Leica VT1200S vibratome and permeabilized with 0.3% Triton X-100 for 2 hours. Sections were blocked with 10% goat serum and 0.1% sodium azide in 1% BSA in PBS for 1 hour and incubated with primary antibody for Tubulin βlll (1:200, Millipore Sigma, catalog # T5076) overnight on a shaker at room temperature. Alexa Fluor 568 conjugated secondary antibody (1:200 Thermo Fisher, catalog A11019) was used to detect Tubulin βlll primary antibody and incubated with sections for 2 hours on a shaker at room temperature. After washing in PBS, the sections were incubated with primary antibody for p-eEF2 (1:100, Cell Signaling, catalog # 2331) overnight on a shaker at room temperature. Alexa Fluor 488 conjugated secondary antibody (1:200 Thermo Fisher, catalog A11008) was used to detect p-eEF2 primary antibody and incubated with sections for 2 hours on a shaker at room temperature. The sections were then mounted with medium containing DAPI (Vector labs, H-1200) and imaged using an Olympus FV1200 confocal microscope. All parameters (pinhole, contrast, gain, and offset) were held constant for all sections across the same experiment.

**Golgi staining**

Mouse brains were processed using the FD Rapid Golgi Stain Kit in accordance with the manufacturer's instructions (FD Neurotechnologies, catalog # PK401). Transverse sections (100 μm) were prepared using a Leica VT1200S vibratome and mounted onto gelatin-coated slides. Development of silver staining was performed according to kit instructions. Sections were dehydrated through a graded ethanol series and cleared in xylene. Slides were coverslipped with mounting medium (Vector Labs, catalog # H-5000) and area CA1 stratum radiatum apical dendrites were imaged at 100X on a Keyence BZ-X710 microscope. 3 mice were used in each group, around 12 slices were prepared for each mouse, and around 3dendrites were counted for each slice. For spine analysis, images were blinded to the experimenters, and spines were manually counted and sorted as previously described(*40*).

**Transmission electron microscopy (TEM)**

Mouse brains were cut into 1-mm-thick transverse slices using a Leica VT1200S vibratome. About 1mm^3^ tissue from CA1 area of hippocampus was dissected and immediately fixed in 2.5% glutaraldehyde/1% paraformaldehyde in 0.1 M Millonig's phosphate buffer (pH 7.3) overnight. The samples were washed in buffer and post-fixed with 1% osmium tetroxide in phosphate buffer for 1 hour. After washing, samples were dehydrated through a graded series of ethanol solutions. For the preparation of resin infiltration, the samples were incubated in propylene oxide for two 15min changes. Finally, the samples were gradually infiltrated with 1:1, 1:2, and pure solutions of Spurr's resin and cured in a 70°C oven overnight. Sections of 90 nm were cut using a Reichert-Jung Ultra-cut E ultramicrotome, stained with lead citrate and uranyl acetate, and viewed with a Tecnai Spirit transmission electron microscope operating at 80 kV (FEI Co.). Images were obtained with a 2Vu CCD camera (Advanced Microscopy Techniques) at 13,000X. 3 mice were used in each group, and 10 images were taken for each mouse. Analysis for PSDs was performed as previously described(*64*). Samples and images were blinded to experimenters.

**LC-MS/MS**

Mouse hippocampi were dissected, flash-frozen on dry ice and sent to Proteomics Core at Rutgers University. Tissues were lysed in PBS with protease/phosphatase inhibitors using a bead mill homogenizer. Tubes were centrifuged at 18000g for 10 minutes, and the supernatant was used for analysis. Protein concentration was determined by BCA analysis, and 50µg of protein was loaded to 10% Bis-Tris gel. The gel slices were then incubated at 60˚C for 30 min with 10 mM DTT. After cooling to room temperature, 20 mM iodoacetamide were added and kept in the dark for 1 hour to block free cysteine. The samples were digested by trypsin at 1:50 (w:w, trypsin:sample) and incubated at 37˚C overnight. The digested peptides were extracted and dried under vacuum and washed with 50% acetonitrile to pH neutral. Samples were then labeled with 16-plex label reagent (TMTpro, Thermo Scientific, catalog # A44520) following the manufacture’s protocol. Labeled samples were pooled at 1:1 ratio for a small volume and analyzed with LC-MS/MS to get normalization factor. The labeling efficiency is 97.4% for the cohort. The pooled samples were dried and desalted with SPEC Pt C18 (Agilent Technologies, catalog # A57203). The desalted samples were fractionated using Agilent 1100 series. The samples were solubilized in 200 µl of 20 mM ammonium (pH 10), and injected onto an Xbridge column (Waters, C18 3.5 µm 2.1X150 mm) using a linear gradient of 1% B/min from 2-45% of B (buffer A: 20 mM ammonium, pH 10, B: 20 mM ammonium in 90% acetonitrile, pH 10). UV 214 was monitored. 1min fractions were collected. Selected fractions were desalted with stage tip and analyzed by LC-MS/MS. LC-MS/MS was performed using a Dionex rapid-separation liquid chromatography system interfaced with an Eclipse (Thermo Scientific). Selected desalted fractions were loaded onto an Acclaim PepMap 100 trap column (75µm x 2cm, Thermo Scientific) and washed with Buffer A (0.1% trifluoroacetic acid) for 5min with a flow rate of 5µl/min. The trap was brought in-line with the nano analytical column (nanoEase, MZ peptide BEH C18, 130A, 1.7µm, 75µmx20cm, Waters) with flow rate of 300 nl/min with a multistep gradient (4% to 15% buffer B [0.16% formic acid and 80% acetonitrile] in 20min, then 15%–25% B in 40min, followed by 25%–50% B in 30min). The scan sequence began with an MS1 spectrum (Orbitrap analysis, resolution 120,000, scan range from 350–1600 Th, automatic gain control (AGC) target 1E6, maximum injection time 100 ms). For SPS3, MSMS analysis consisted of collision-induced dissociation (CID), quadrupole ion trap analysis, AGC 2E4, normalized collision energy (NCE) 35, maximum injection time 55ms, and isolation window at 2Da. Following acquisition of each MS2 spectrum, an MS3 spectrum in which 10 MS2 fragment ions were captured in the MS3 precursor population using isolation waveforms with multiple frequency notches. MS3 precursors were fragmented by HCD and analyzed using the Orbitrap (NCE 55, AGC 1.5E5, maximum injection time 150ms, resolution was 50,000 at 400 Th, scan range 100-500). The whole cycle is repeated for 3s before repeat from an MS1 spectrum. Dynamic exclusion of 1 repeat and duration of 60s was used to reduce the repeat sampling of peptides. LC-MS/MS data were analyzed with Proteome Discoverer 2.4 (Thermo Scientific) with sequence search engine against uniprot mouse proteome reference database and a database consisted of common lab contaminants. The MS mass tolerance was set at +/- 10 ppm, MSMS mass tolerance were set at +/- 0.4Da for proteome. TMTpro on K and N-terminus of peptides and carbamiodomethyl on cysteine was set as static modification. Methionine oxidation, protein N-terminal acetylation, N-terminal methionine loss or N-terminal methionine loss plus acetylation were set as dynamic modifications for proteome data. Percolator was used for results validation. Concatenated reverse database was used for target-decory strategy. High confidence for protein and peptides were defined as FDR <0.01, medium confidence was defined as FDR <0.05. For reporter ion quantification, reporter abundance was set to use signal/noise ratio (S/N) if all spectrum files have S/N values. Otherwise, intensities were used. Quan value was corrected for isotopic impurity of reporter ions. Co-isolation threshold was set at 75%. Average reporter S/N threshold was set at 10. SPS mass matches % threshold was set at 65%. Protein abundance of each channel was calculated using summed S/N of all unique+razor peptide. The abundance was further normalized to summed abundance value for each channel over all peptides identified within a file.

**Table.S1.** **Demographic information of control and DS patients**

| **Code** | **Age (year)** | **Sex** | **Diagnosis** | **RIN** | **PMI (hour)** |
| --- | --- | --- | --- | --- | --- |
| 5439 | 57 | male | DS | 7.4 | 3 |
| 5510 | 65 | male | DS, AD | 8.5 | 10 |
| 5600 | 57 | female | DS, AD | 7.9 | 6 |
| 3717 | 57 | male | DS, Seizure | NA | 22 |
| 4395 | 56 | male | DS, AD | NA | 14.7 |
| 1206 | 57 | male | control | 7.1 | 16 |
| 4238 | 55 | female | control | 6.5 | 12 |
| 5656 | 51 | female | Control | 8.4 | 21 |
| 5697 | 67 | male | control | 8 | 15 |
| 5862 | 58 | male | control | 8 | 26 |

PMI Postmortem interval, RIN RNA Integrity Number


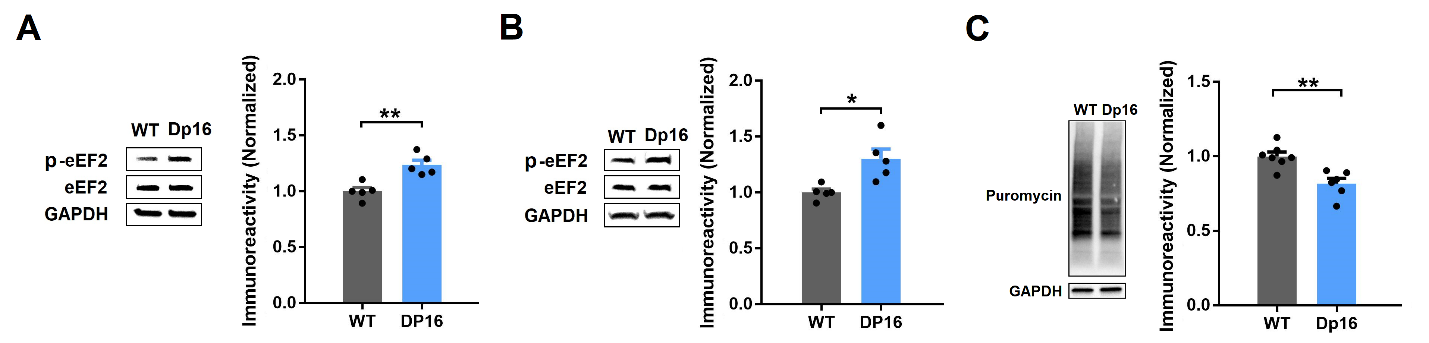


**Fig.S1.** **eEF2K signaling is dysregulated in the hippocampi of Dp16 mice**

**(A)** Representative western blot images of p-eEF2, eEF2 and GAPDH in hippocampal whole lysates from WT and Dp16 mice, and quantification of p-eEF2 levels in the two groups. n=5 in each group. ** p<0.01, t=4.463, df=8, unpaired t test. (**B)** Representative western blot images of p-eEF2, eEF2 and GAPDH in hippocampal synaptosomes from WT and Dp16 mice, and quantification of p-eEF2 levels in the two groups. n=5 in each group. * p<0.05, t=3.293, df=8, unpaired t test. (**C)** Representative images and quantification of the SUnSET assay in hippocampal slices from WT and Dp16 mice. WT n=7; Ts65Dn n=6. ** p<0.01, t=4.012, df=11, unpaired t test.

**
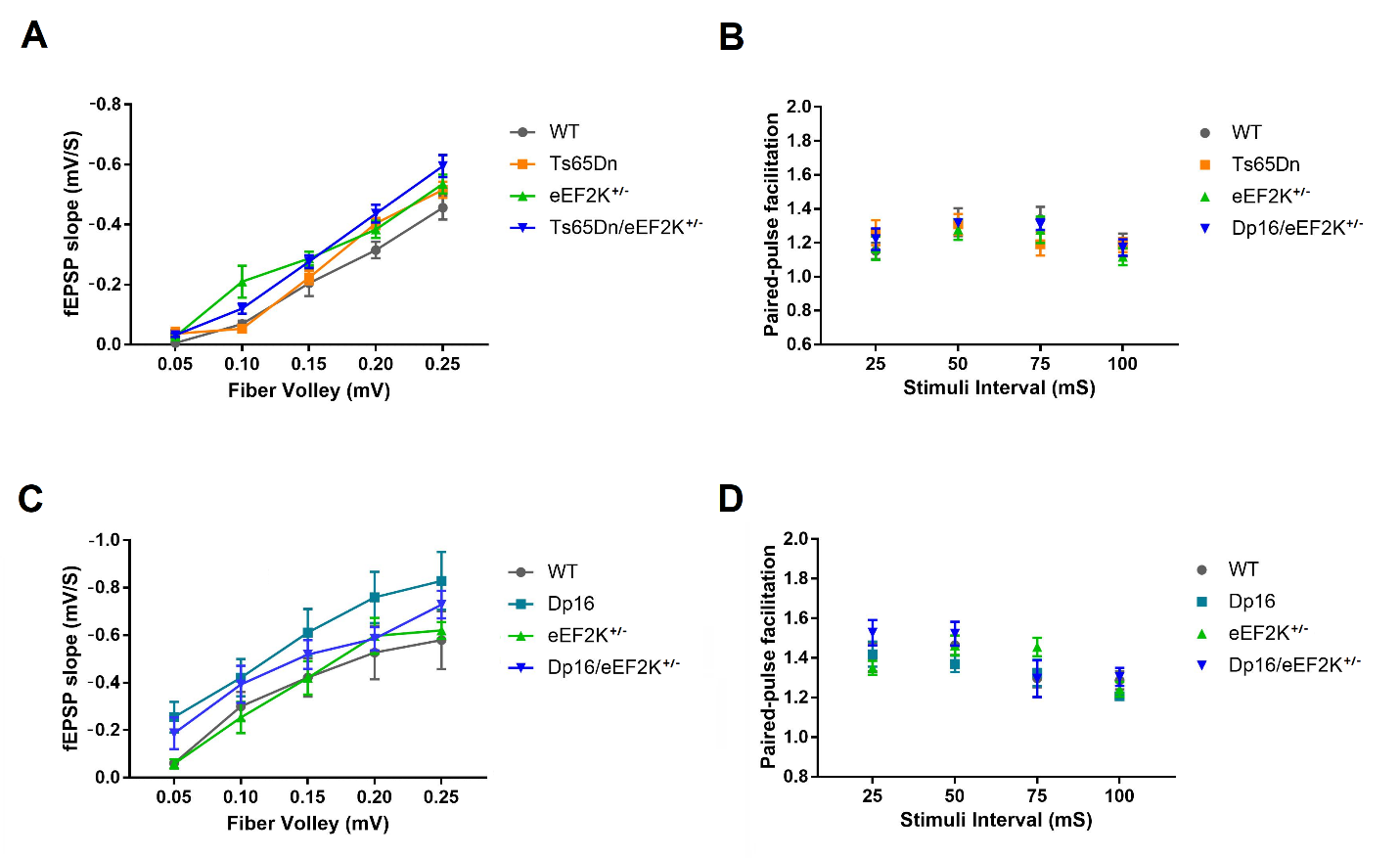
**

**Fig.S2.** **Analysis of the I/O relationship and PPF performance in the two DS mouse models with suppression of eEF2K**

**(A)** I/O relationship in WT, Ts65Dn, eEF2K^+/-^ and Ts65Dn/eEF2K^+/-^ mice. WT n=10; Ts65Dn n=8; eEF2K^+/-^ n=10; Ts65Dn/eEF2K^+/-^ n=8. Interaction F(9, 128)=0.5178, p=0.8595; intensity factor F(3, 128)=4.318, p=0.0062; genotype factor F(3, 128)=0.4213, p=0.7380; two-way ANOVA with Tukey’s post-hoc test. (**B)** PPF in WT, Ts65Dn, eEF2K^+/-^ and Ts65Dn/eEF2K^+/-^ mice. WT n=10; Ts65Dn n=8; eEF2K^+/-^ n=10; Ts65Dn/eEF2K^+/-^ n=8. Interaction F(12, 160)=1.636, p=0.0865; time factor F(4, 160)=203.5, p<0.0001; genotype factor F(3, 160)=10.00, p<0.0001; two-way ANOVA with Tukey’s post-hoc test. (**C)** I/O relationship in WT, Dp16, eEF2K^+/-^ and Dp16/eEF2K^+/-^ mice. WT n=8; Dp16 n=9; eEF2K^+/-^ n=8; Dp16/eEF2K^+/-^ n=7. Interaction F(12, 111)=0.1804, p=0.9989; intensity factor F(4, 111)=30.97, p<0.0001; genotype factor F(3, 111)=7.222, p=0.0002; two-way ANOVA with Tukey’s post-hoc test. (**D)** PPF in WT, Dp16, eEF2K^+/-^ and Dp16/eEF2K^+/-^ mice. WT n=10; Dp16 n=10; eEF2K^+/-^ n=8; Dp16/eEF2K^+/-^ n=8. Interaction F(9, 127)=1.255, p=0.2681; time factor F(3, 127)=10.18, p<0.0001; genotype factor F(3, 127)=1.541, p=0.2072; two-way ANOVA with Tukey’s post-hoc test.


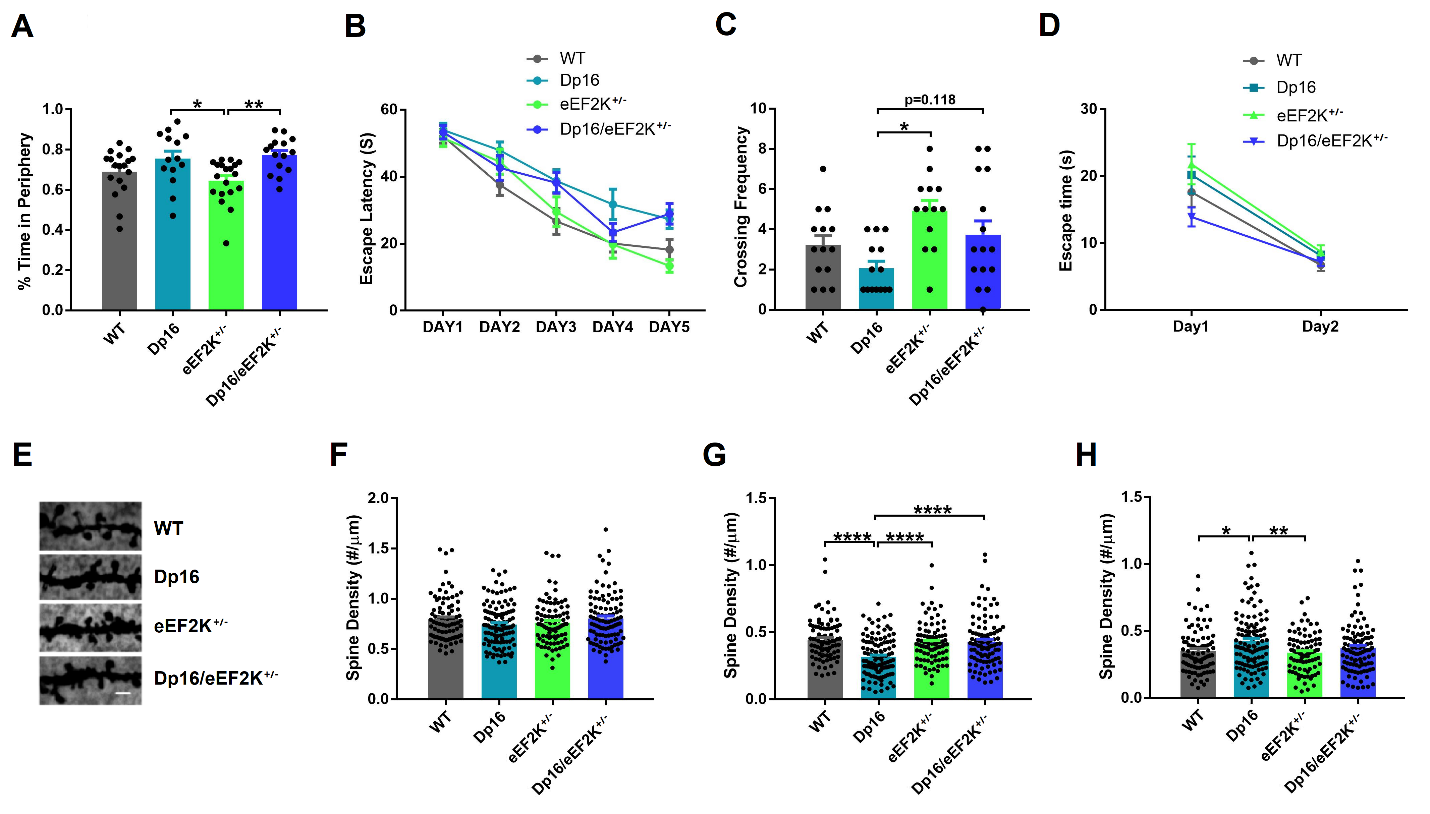


**Fig.S3.** **Suppression of eEF2K alleviates spatial memory deficits and morphological spine abnormalities in Dp16 mice**

**(A)** Ratio of time spent in the periphery in the OF test of WT, Dp16, eEF2K^+/-^ and Dp16/eEF2K^+/-^ mice. WT n=18; Dp16 n=14; eEF2K^+/-^ n=19; Dp16/eEF2K^+/-^ n=15. * p<0.05, ** p<0.01, F(3, 62)=4.657, one-way ANOVA with Tukey’s post-hoc test. (**B)** Escape latencies of WT, Dp16, eEF2K^+/-^ and Dp16/eEF2K^+/-^ mice during 5-days training phase of MWM test. WT n=15; Dp16 n=14; eEF2K^+/-^ n=13; Dp16/eEF2K^+/-^ n=15. (**C)** Frequencies of crossing the “platform” of WT, Dp16, eEF2K^+/-^ and Dp16/eEF2K^+/-^ mice in the probe trial. WT n=14; Dp16 n=14; eEF2K^+/-^ n=13; Dp16/eEF2K^+/-^ n=15. * p<0.05, F(3, 52)=4.894, one-way ANOVA with Tukey’s post-hoc test. (**D)** Escape latencies of WT, Dp16, eEF2K^+/-^ and Dp16/eEF2K^+/-^ mice in the VP test. WT n=15; Dp16 n=14; eEF2K^+/-^ n=14; Dp16/eEF2K^+/-^ n=16. **(E)** Representative Golgi staining images in the CA1 areas of hippocampi from WT, Dp16, eEF2K^+/-^ and Dp16/eEF2K^+/-^ mice. Scale bar=2µm. (**F)** Cumulative data of total spine densities of hippocampal CA1 dendrites from WT, Dp16, eEF2K^+/-^ and Dp16/eEF2K^+/-^ mice. WT n=88 dendrites; Dp16 n=112 dendrites; eEF2K^+/-^ n=88 dendrites; Dp16/eEF2K^+/-^ n=99 dendrites. 3 mice in each group. F(3, 383)=1.746, p=0.1571, one-way ANOVA with Tukey’s post-hoc test. (**G)** Cumulative data of mature spine densities of hippocampal CA1 dendrites from WT, Dp16, eEF2K^+/-^ and Dp16/eEF2K^+/-^ mice. WT n=88 dendrites; Dp16 n=112 dendrites; eEF2K^+/-^ n=88 dendrites; Dp16/eEF2K^+/-^ n=99 dendrites. 3 mice in each group. **** p<0.0001, F(3, 383)=14.60, one-way ANOVA with Tukey’s post-hoc test. (**H)** Cumulative data of immature spine densities of hippocampal CA1 dendrites from WT, Dp16, eEF2K^+/-^ and Dp16/eEF2K^+/-^ mice. WT n=88 dendrites; Dp16 n=112 dendrites; eEF2K^+/-^ n=88 dendrites; Dp16/eEF2K^+/-^ n=99 dendrites. 3 mice in each group. * p<0.05, ** p<0.01, F(3, 383)=4.655, one-way ANOVA with Tukey’s post-hoc test.


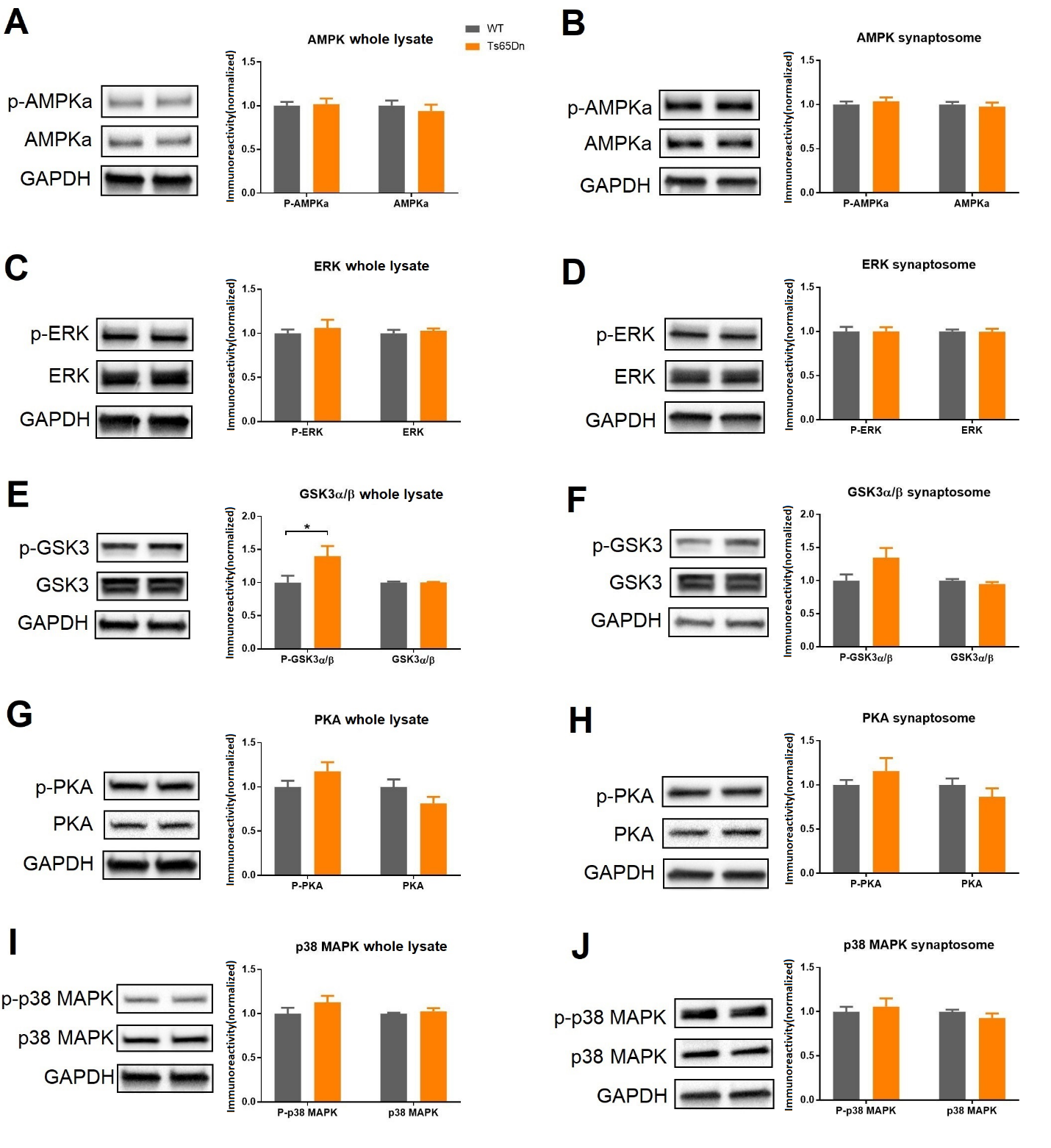


**Fig.S4.** **Assessment of canonical upstream regulators of eEF2K in the hippocampi of Ts65Dn mice**

**(A)** Representative western blot images of p-AMPKα, AMPKα and GAPDH in hippocampal whole lysates from WT and Ts65Dn mice, and quantification of p-AMPKα and AMPKα levels in the two groups. n=8 in each group. p-AMPKα t=0.2240, df=14, p=0.8260; AMPKα t=0.6344, df=14, p=0.5361; unpaired t test. (**B)** Representative western blot images of p-AMPKα, AMPKα and GAPDH in hippocampal synaptosomes from WT and Ts65Dn mice, and quantification of p-AMPKα and AMPKα levels in the two groups. n=8 in each group. p-AMPKα t=0.6371, df=14, p=0.5344; AMPKα t=0.3978, df=14, p=0.6967; unpaired t test. (**C)** Representative western blot images of p-ERK, ERK and GAPDH in hippocampal whole lysates from WT and Ts65Dn mice, and quantification of p-ERK and ERK levels in the two groups. n=8 in each group. p-ERK t=0.6102, df=14, p=0.5515; ERK t=0.5997, df=14, p=0.5583; unpaired t test. (**D)** Representative western blot images of p-ERK, ERK and GAPDH in hippocampal synaptosomes from WT and Ts65Dn mice, and quantification of p-ERK and ERK levels in the two groups. n=8 in each group. p-ERK t=0.0113, df=14, p=0.9912; ERK t=0.0907, df=14, p=0.9290; unpaired t test. (**E)** Representative western blot images of p-GSK3α/β, GSK3α/β and GAPDH in hippocampal whole lysates from WT and Ts65Dn mice, and quantification of p-GSK3α/β and GSK3α/β levels in the two groups. n=8 in each group. * p<0.05, p-GSK3α/β t=2.184, df=14, p=0.0465; GSK3α/β t=0.1267, df=14, p=0.9010; unpaired t test. (**F)** Representative western blot images of p-GSK3α/β, GSK3α/β and GAPDH in hippocampal synaptosomes from WT and Ts65Dn mice, and quantification of p-GSK3α/β and GSK3α/β levels in the two groups. n=8 in each group. p-GSK3α/β t=2.041, df=14, p=0.0605; GSK3α/β t=1.335, df=14, p=0.2033; unpaired t test. (**G)** Representative western blot images of p-PKA, PKA and GAPDH in hippocampal whole lysates from WT and Ts65Dn mice, and quantification of p-PKA nad PKA levels in the two groups. n=8 in each group. p-PKA t=1.424, df=14, p=0.1764; PKA t=1.649, df=14, p=0.1215; unpaired t test. (**H)** Representative western blot images of p-PKA, PKA and GAPDH in hippocampal synaptosomes from WT and Ts65Dn mice, and quantification of p-PKA and PKA levels in the two groups. n=8 in each group. p-PKA t=0.9868, df=14, p=0.3405; PKA t=1.110, df=14, p=0.2858; unpaired t test. (**I)** Representative western blot images of p-p38 MAPK, p38 MAPK and GAPDH in hippocampal whole lysates from WT and Ts65Dn mice, and quantification of p-p38 MAPK and p38 MAPK levels in the two groups. n=8 in each group. p-p38 MAPK t=1.307, df=14, p=0.2123; p38 MAPK t=0.7414, df=14, p=0.4707; unpaired t test. (**J)** Representative western blot images of p-p38 MAPK, p38 MAPK and GAPDH in hippocampal synaptosomes from WT and Ts65Dn mice, and quantification of p-p38 MAPK and p38 MAPK levels in the two groups. n=8 in each group. p-p38 MAPK t=0.5367, df=14, p=0.5999; p38 MAPK t=1.245, df=14, p=0.2337; unpaired t test.


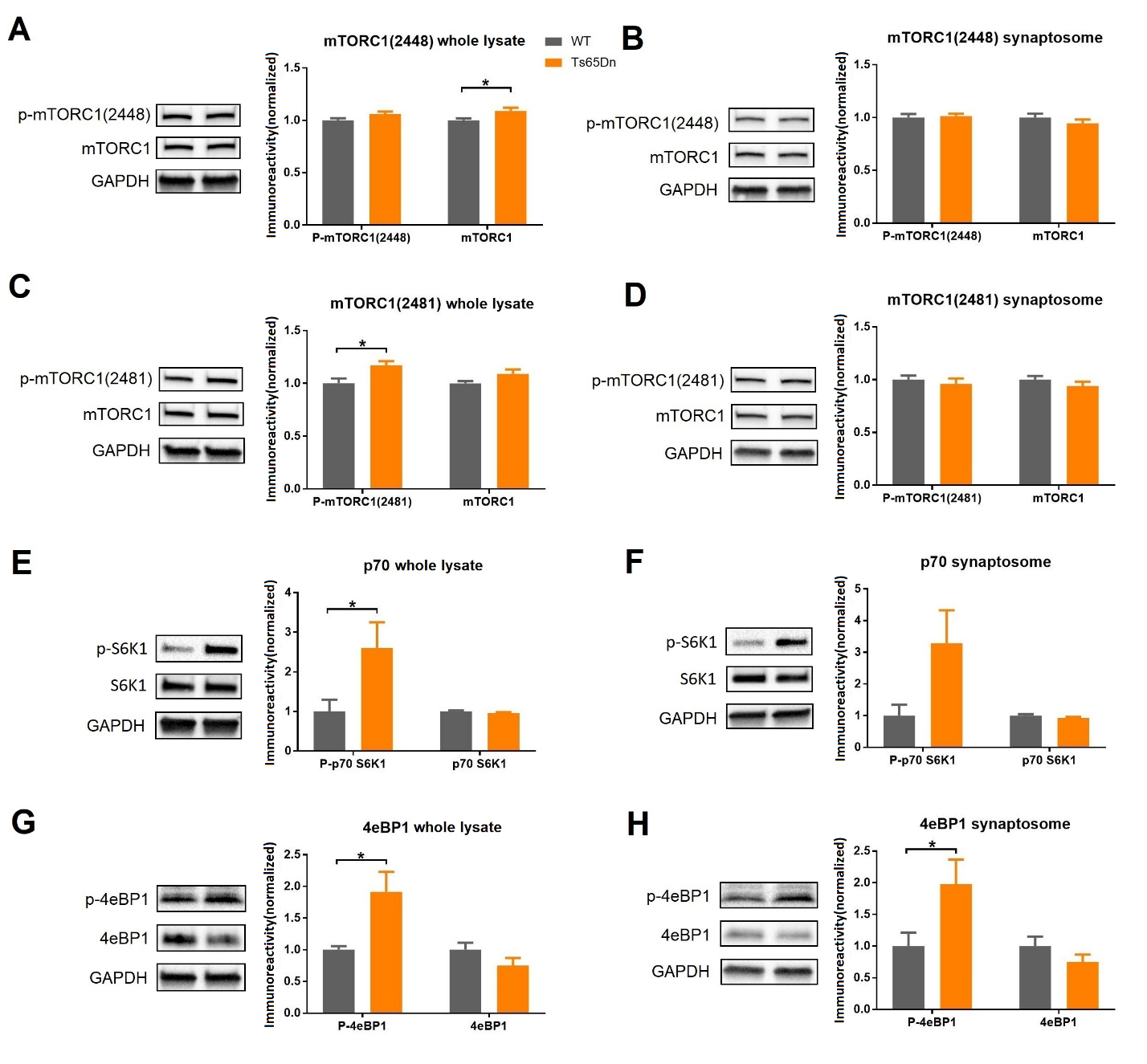


**Fig.S5.** **Assessment of potential upstream regulators of eEF2K in the hippocampi of Ts65Dn mice**

**(A)** Representative western blot images of p-mTORC1(2448), mTORC1 and GAPDH in hippocampal whole lysates from WT and Ts65Dn mice, and quantification of p-mTORC1(2448) and mTORC1 levels in the two groups. n=8 in each group. * p<0.05, p-mTORC1(2448) t=0.6102, df=14, p=0.0784; mTORC1 t=2.454, df=14, p=0.0278; unpaired t test. (**B)** Representative western blot images of p-mTORC1(2448), mTORC1 and GAPDH in hippocampal synaptosomes from WT and Ts65Dn mice, and quantification of p-mTORC1(2448) and mTORC1 levels in the two groups. n=8 in each group. p-mTORC1(2448) t=0.3406, df=14, p=0.7385; mTORC1 t=1.058, df=14, p=0.3080; unpaired t test. (**C)** Representative western blot images of p-mTORC1(2481), mTORC1 and GAPDH in hippocampal whole lysates from WT and Ts65Dn mice, and quantification of p-mTORC1(2481) and mTORC1 levels in the two groups. n=8 in each group. * p<0.05, p-mTORC1(2481) t=2.862, df=14, p=0.0126; mTORC1 t=1.953, df=14, p=0.0711; unpaired t test. (**D)** Representative western blot images of p-mTORC1(2481), mTORC1 and GAPDH in hippocampal synaptosomes from WT and Ts65Dn mice, and quantification of p-mTORC1(2481) and mTORC1 levels in the two groups. n=8 in each group. p-mTORC1(2481) t=0.6149, df=14, p=0.5485; mTORC1 t=1.115, df=14, p=0.2836; unpaired t test. (**E)** Representative western blot images of p-S6K1, S6K1 and GAPDH in hippocampal whole lysates from WT and Ts65Dn mice, and quantification of p-S6K1 and S6K1 levels in the two groups. n=8 in each group. * p<0.05, p-S6K1 t=2.243, df=14, p=0.0416; S6K1 t=1.002, df=14, p=0.3333; unpaired t test. (**F)** Representative western blot images of p-S6K1, S6K1 and GAPDH in hippocampal synaptosomes from WT and Ts65Dn mice, and quantification of p-S6K1 and S6K1 levels in the two groups. n=8 in each group. p-S6K1 t=2.091, df=14, p=0.0552; S6K1 t=1.195, df=14, p=0.2519; unpaired t test. (**G)** Representative western blot images of p-4eBP1, 4eBP1 and GAPDH in hippocampal whole lysates from WT and Ts65Dn mice, and quantification of p-4eBP1 and 4eBP1 levels in the two groups. n=8 in each group. * p<0.05, p-4eBP1 t=2.858, df=14, p=0.0127; 4eBP1 t=1.536, df=14, p=0.1469; unpaired t test. (**H)** Representative western blot images of p-4eBP1, 4eBP1 and GAPDH in hippocampal synaptosomes from WT and Ts65Dn mice, and quantification of p-4eBP1 and 4eBP1 levels in the two groups. n=8 in each group. * p<0.05, p-4eBP1 t=2.200, df=14, p=0.0451; 4eBP1 t=1.312, df=14, p=0.2107; unpaired t test.

**
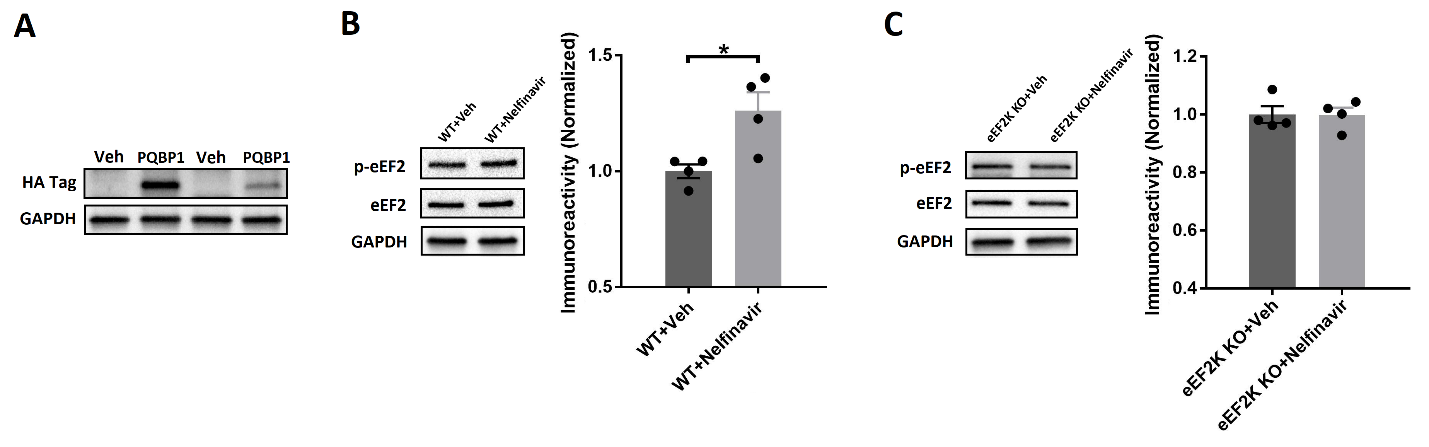
**

**Fig.S6.** **Viral overexpression of PQBP1 and increased eEF2 phosphorylation through eEF2K by nelfinavir treatment in the hippocampi of mice**

**(A)** Western blot images of HA tag and GAPDH in the hippocampal tissues from WT mice injected with either vehicle or PQBP1 virus. n=2 in each group. (**B)** Representative western blot images of p-eEF2, eEF2 and GAPDH in the hippocampal slices from WT mice treated with either vehicle or nelfinavir, and quantification of p-eEF2 levels in the two groups. n=4 in each group. * p<0.05, t=3.112, df=6, unpaired t test. (**C)** Representative western blot images of p-eEF2, eEF2 and GAPDH in the hippocampal slices from eEF2K knockout (KO) mice treated with either vehicle or nelfinavir, and quantification of p-eEF2 levels in the two groups. n=4 in each group. t=0.0356, df=6, p=0.9727, unpaired t test.


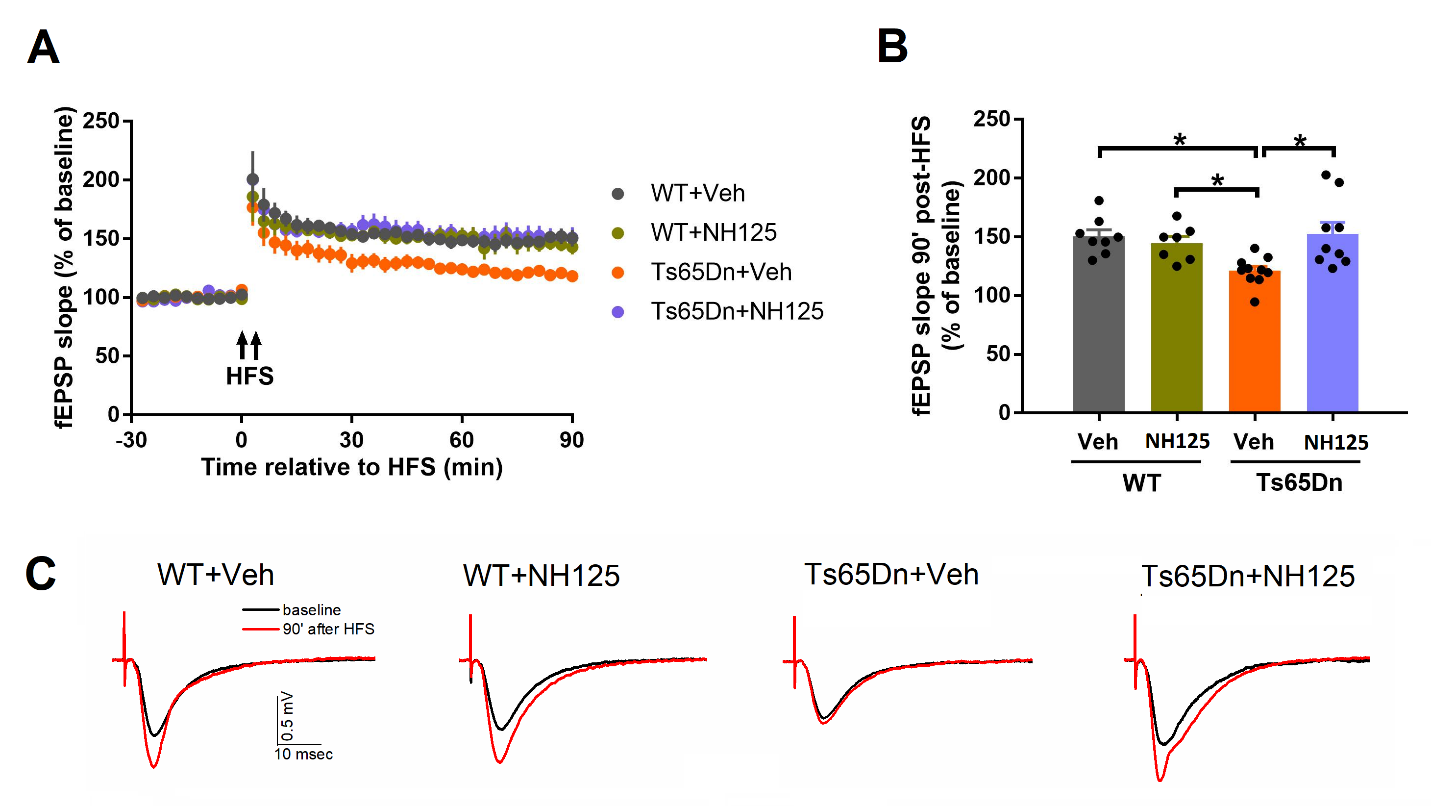


**Fig.S7.** **LTP studies in WT and Ts65Dn hippocampal slices treated with NH125**

A. LTP in WT+vehicle, WT+NH125, Ts65Dn+vehicle, and Ts65Dn+NH125 hippocampal slices. Arrows indicate HFS. WT+vehicle n=8; WT+NH125 n=7; Ts65Dn+vehicle n=10; Ts65Dn+NH125 n=9. **B.** Cumulative data showing fEPSP slopes at 90 min after HFS in WT+vehicle, WT+NH125, Ts65Dn+vehicle, and Ts65Dn+NH125 hippocampal slices. WT+vehicle n=8; WT+NH125 n=7; Ts65Dn+vehicle n=10; Ts65Dn+NH125 n=9. * p<0.05, F(3, 30)=5.328, one-way ANOVA with Tukey’s post-hoc test. **C.** Representative traces before and after HFS in WT+vehicle, WT+NH125, Ts65Dn+vehicle, and Ts65Dn+NH125 hippocampal slices.
